# Supplementary material for: A strong sense of coherence associated with reduced risk of anxiety disorder among women in disadvantaged circumstances: British population study
Source: BMJ Open. 2018 Apr 23;8(4):e018501. doi: 10.1136/bmjopen-2017-018501 (PMC5914722; doi:10.1136/bmjopen-2017-018501)
Supplement: Supplementary file 1 [file bmjopen-2017-018501supp001.pdf]

Appendix 1: Characteristics of participants who consented (n=30,445) and refused (n=43,452) to take part in the EPIC-Norfolk cohort study

	Percentage (number)	
**Characteristic**	**Consented**	**Did not consent**
**Age**		
<50	27.5 (8366)	33.7 (14647)
50-60	30.3 (9230)	29.5 (12819)
60-70	32.5 (9879)	27.4 (11898)
>=70	9.8 (2970)	9.4 (4088)
**Gender**		
Female	55.0 (16744)	49.0 (21296)
Male	45.0 (13701)	51.0 (22156)

Appendix 2 – Supplementary Material

We imputed missing data separately for women with a weak and strong sense of coherence (SOC). Based on the literature, we identified and retained seven auxiliary variables. Our imputation model included all variables in the analysis model and the auxiliary variables.

To retain as much information as possible, we conducted the imputations on non-transformed data-the original variables in our dataset. We imputed data using the fully conditional specification, and specified a linear regression model for continuous data that were normally distributed; predictive mean matching for continuous data that were not normally distributed; logistic regression for binary and ordinal variables; and the discrim function for nominal variables. Variable estimates were subsequently averaged from 5 imputed datasets using Rubin's rules (we transformed the data before running the analytic model of interest within each of the imputed datasets).[1] Because the intra-class correlation was found to be negligible, we used logistic regression for the final model using imputed data.

We checked whether the imputations were acceptable by comparing 1) the means and standard deviations of recorded and imputed values for continuous variables, and 2) the frequencies and percentages of recorded and imputed values for each level of categorical variables.

Analyses were done using SAS 9.3 and p-values less than 0.05 were considered statistically significant.

Auxiliary variables used in the imputation model

Variable	Questionnaire	Description of variable
**Psychological factors**		
Paternal affection	Health and Life Experiences Questionnaire (HLEQ)	Self-reported paternal affection using the Rossi scale; this includes components such as quality of relationship with the father, among other items.[2]
Mastery	Health and Life Experiences Questionnaire (HLEQ)	Self-reported using the Pearlin and Schooler Mastery Scale. Mastery is a coping resource and refers to the degree to which one has control over one's life.[3,4]
Neuroticism	Health and Life Experiences Questionnaire (HLEQ)	Self-reported using the Eysenck Personality Inventory. Neuroticism refers to a proneness to experiencing negative emotions.[5]
**Sociodemographic factors**		
School age	Health and Lifestyle (HLQ) Questionnaire	Self-reported age when participant left school.
**Physical health**		

Systolic blood pressure	Baseline health check	Systolic blood pressure measured using an Accutorr noninvasive oscillometric blood pressure monitor.
Diastolic blood pressure	Baseline health check	Diastolic blood pressure measured using an Accutorr noninvasive oscillometric blood pressure monitor.
History of high cholesterol	Health and Lifestyle Questionnaire (HLQ)	Self-reported history of high cholesterol

References

1. Berglund P, Heeringa S. Multiple imputation of missing data using SAS. Cary, NC: *SAS Institute Inc*, 2014.
2. Rossi AS. Caring and doing for others: social responsibility in the domains of family, work, and community. Chicago: *University of Chicago Press*, 2001.
3. Pearlin LI, Menaghan EG, Lieberman MA, Mullan JT. The stress process. *J Health Soc Behav* 1981;22:337-56.
4. Pearlin LI, Schooler C. The structure of coping. *J Health Soc Behav* 1978;19:2-21.
5. Shipley BA, Weiss A, Der G, Taylor MD, Deary IJ. Neuroticism, extraversion, and mortality in the UK Health and Lifestyle Survey: a 21-year prospective cohort study. *Psychosom Med* 2007;69:923-31 doi: 10.1097/PSY.0b013e31815abf83 [published Online First 8 November 2007].
