# Supplementary material for: A strong sense of coherence associated with reduced risk of anxiety disorder among women in disadvantaged circumstances: British population study
Source: BMJ Open. 2018 Apr 23;8(4):e018501. doi: 10.1136/bmjopen-2017-018501 (PMC5914722; doi:10.1136/bmjopen-2017-018501)
Supplement: Author's manuscript [file bmjopen-2017-018501.draft_revisions.pdf]

BMJ Open is committed to open peer review. As part of this commitment we make the peer review history of every article we publish publicly available.

When an article is published we post the peer reviewers' comments and the authors' responses online. We also post the versions of the paper that were used during peer review. These are the versions that the peer review comments apply to.

The versions of the paper that follow are the versions that were submitted during the peer review process. They are not the versions of record or the final published versions. They should not be cited or distributed as the published version of this manuscript.

BMJ Open is an open access journal and the full, final, typeset and author-corrected version of record of the manuscript is available on our site with no access controls, subscription charges or pay-per-view fees (http://bmjopen.bmj.com).

If you have any questions on BMJ Open's open peer review process please email
info.bmjopen@bmj.com

BMJ Open

Sense of coherence as a coping mechanism for women with anxiety living in deprivation: British population study

Journal:	*BMJ Open*
Manuscript ID	bmjopen-2017-018501
Article Type:	Research
Date Submitted by the Author:	04-Jul-2017
Complete List of Authors:	Remes, Olivia; University of Cambridge, Public Health and Primary Care<br>Wainwright, Nicholas; University of Cambridge, UK, Public Health and Primary Care (retired)<br>Surtees, Paul; University of Cambridge, Department of Public Health and Primary Care<br>LaFortune, Louise; University of Cambridge, Institute of Public Health<br>Khaw, Kay-Tee; University of Cambridge, Department of Public Health and Primary Care<br>Brayne, Carol; University of Cambridge, Institute of Public Health
**Primary Subject Heading**:	Mental health
Secondary Subject Heading:	Epidemiology, Public health
Keywords:	EPIDEMIOLOGY, MENTAL HEALTH, Anxiety disorders < PSYCHIATRY, PUBLIC HEALTH

SCHOLARONE™
Manuscripts

Sense of coherence as a coping mechanism for women with anxiety living in deprivation: British population study

Olivia Remes[1], Nick Wainwright[2], Paul Surtees[1], Louise Lafortune[1], Kay-Tee Khaw[3], Carol Brayne[1]

[1]Department of Public Health and Primary Care, University of Cambridge, Forvie Site, Robinson Way, Cambridge, UK CB2 0SR

[2](Retired) Department of Public Health and Primary Care, University of Cambridge, 2 Worts' Causeway, Cambridge, UK CB1 8RN

[3]Department of Public Health and Primary Care, University of Cambridge, 2 Worts' Causeway, Cambridge, UK CB1 8RN

Figures: 1; Tables: 3; Appendix: 2

Correspondence to: Olivia Remes or260@medschl.cam.ac.uk, telephone: 0777674550

For peer review only

Abstract

OBJECTIVE

Many patients receiving medical treatment for anxiety relapse or do not improve. Research has therefore been turning to coping mechanisms as a way to decrease anxiety rates. Previously, we showed that living in a deprived area significantly increases the risk of generalized anxiety disorder (GAD) in women, but not in men. The objective of this study will be to assess whether sense of coherence (coping mechanism) buffers the influence of area deprivation on women's risk of GAD using data from the European Prospective Investigation of Cancer-Norfolk.

DESIGN

Large, population study.

SETTING

UK population-based cohort.

PARTICIPANTS

30,445 people over the age of 40 were recruited through general practice registers in England. Of these, 20,921 completed a structured health and lifestyle questionnaire used to assess generalised anxiety disorder and sense of coherence. Area deprivation was measured using 1991 Census data, and sense of coherence and anxiety were examined in 1996-2000. 10,277 women had complete data on all covariates.

MAIN OUTCOME MEASURE

Past-year generalised anxiety disorder defined according to the Diagnostic and Statistical Manual of Mental Disorders, fourth edition.

RESULTS

In this study, 2.5% (261/10,277) of women had generalized anxiety disorder. In those with a strong sense of coherence, area deprivation was not significantly associated with anxiety (OR=1.42, 95% CI: 0.86, 2.37). However, among women with a weak sense of coherence, those living in deprived areas had an 88% higher likelihood of having generalized anxiety disorder compared to those living in more affluent areas (OR=1.88, 95% CI: 1.28, 2.77).

CONCLUSION

The absolute number of women living in deprived conditions is large worldwide, and significant numbers are affected by generalized anxiety disorder. Sense of coherence moderates the association between area deprivation and anxiety in women; therefore, interventions targeting coping mechanisms may need to be considered for people with anxiety.

Key words: Anxiety, anxiety disorders, risk factors, gender

For peer review only

Article summary

Strengths and limitations of this study

- We used a large, population-based sample of middle- and older-aged adults and adjusted for a range of important confounders, such as, sociodemographic factors and medical history.
- We used a structured, self-reported questionnaire to assess presence of past-year GAD, and sense of coherence.
- We measured area deprivation and sense of coherence by using common, valid and theoretically-sound indices.
- Those who participated in this study were somewhat less deprived and healthier than individuals living in other parts of England; therefore, our results may not generalize to people living in extremely deprived circumstances.

For peer review only

Introduction

Generalized anxiety disorder[1] is one of the most common anxiety disorders in the general population.[2-4] It is characterized by excessive and pervasive worry about a number of areas of life, and associated symptoms, such as, restlessness, irritability, muscle tension, sleep difficulties, and concentration problems.[1] If left untreated, this disorder can increase the risk for disability, impairment, and suicide.[2-5] Although treatment for anxiety exists in the form of psychotherapy and pharmacotherapy, very few people who need treatment actually receive it.[6] One of the reasons for this is that physicians under- and misdiagnose those affected, and few people experiencing symptoms seek help from the clinician.[7] Low rates of help-seeking is a result of low general awareness about the disorder and treatment options, and people perceiving their anxiety to be an intractable personality trait, rather than a condition that can be treated. These problems are further compounded by the fact that even after patients are treated, many relapse, while some do not experience improvement in symptoms.[7]

While it is not known what causes anxiety, most studies on risk have focused on individual-level determinants of anxiety disorders such as personal income, education and history of psychopathology.[8-11] However, research has shown that the environment can have a profound effect on mental health, over and above individual-level circumstances. The living context, such as, living in a deprived area, can have harmful effects for mental health independently of personal socio-economic status and lifestyle factors.[12,13] Women have been reported to be particularly affected by their context or the environment in which they are living.[14,15] Women living in poor areas seem to be disproportionately affected by mental disorders.[16, 17]

For peer review only

To reduce the risk of mental disorders among those exposed to disadvantage or adversity, coping skills need to be considered. In particular, sense of coherence, which is a way of viewing life as predictable, manageable, and meaningful, can lower the risk for poor health outcomes.[18]

Two systematic reviews[18,19] showed that SOC is linked to quality of life. A strong SOC is related to good physical and self-perceived health, and is negatively associated with anxiety, depression, and post-traumatic stress disorder (PTSD).[19] In the European-Prospective Investigation of Cancer-Norfolk (EPIC-Norfolk) study of over 18,000 people, a strong SOC contributed to a 20% reduction in all-cause mortality in adults.[20] SOC has also been shown to moderate the influence of disadvantage on mental health outcomes. In a study of people who had faced early childhood deprivation and trauma during the Holocaust, sense of coherence moderated the association between early-life deprivation and posttraumatic stress in old age.[21] A strong SOC can therefore be a major coping resource for preserving health.

Previously[16], we have shown that women living in deprived areas were at increased risk for generalized anxiety disorder. The stress of living in deprivation was harmful for women's mental health, while this association with deprivation was not apparent in men. For this reason, this study will focus on women. The objective of this study will be to determine whether SOC moderates the link between area deprivation and generalized anxiety disorder in women using a large, longitudinal, population cohort.

For peer review only

Methods

Study population

Data were drawn from the population-based European Prospective Investigation of Cancer (EPIC)-Norfolk, described in detail elsewhere[23]. Between 1993 and 1997, 30,445 participants over the age of 40-74 years living in Norwich and the surrounding towns and rural areas were identified through general practice age-sex registers (77,630 people were initially invited to join EPIC-Norfolk). At baseline (1993-97), 30,445 participants consented to join the study and completed a postal Health and Lifestyle (HLQ) questionnaire that captured information on sociodemographics, including age, sex, marital status, highest educational attainment, socio-economic status, and self-reported physician diagnoses of physical diseases. Using participants' postal codes, a measure of area deprivation was derived based on the 1991 Census. Between 1993 and 2000, participants completed self-reported postal questionnaires provided they: 1) were still alive, 2) did not ask to be removed from the study's mailing list, and 3) had a valid mailing address.

All participants recruited through general-practice registers and who completed a baseline health questionnaire were eligible to be included in our study; those who completed a psychosocial questionnaire during follow-up were eligible to be included in our analysis.

Assessment of generalized anxiety disorder (GAD) – outcome

In 1996-2000, 20,921 men and women completed a Health and Life Experiences Questionnaire (HLEQ) used to derive a diagnosis of DSM-IV generalized anxiety disorder (GAD). The primary outcome in this study was past-year GAD. The HLEQ captured the onset

For peer review only

and offset timings of episodes of GAD.[24] Past-year GAD consisted of at least one episode that had offset within 12 months of administration of the HLEQ. DSM-IV GAD was diagnosed if participants reported having uncontrollable, excessive worry for six months or longer on most days than not that resulted in disability or impairment. In addition, at least three of the following symptoms needed to have been present: restlessness, irritability, muscle tension, fatigue, trouble concentrating because of worry, mind going blank, trouble falling asleep, trouble staying asleep, and feeling keyed up or on edge. Of those who completed the HLEQ, 462 met criteria for past-year DSM-IV GAD.

Assessment of potential confounders

Covariates were chosen a priori based on previous literature. The baseline Health and Lifestyle Questionnaire (HLQ) was used to ascertain age, sex, education (highest level of education attained: no qualifications, educated to age 16 years, educated to age 18 years, or educated to degree level), marital status (single, married, widowed, separated, divorced), social class (professionals, managerial and technical occupations, skilled workers divided into non-manual and manual, partly skilled workers and unskilled manual workers), and self-reported physician diagnoses of major medical conditions (self-reported stroke, myocardial infarction, and cancer).

The HLEQ was used to determine presence of DSM-IV major depressive disorder. Disability measures based on the SF-36 were also derived for participants using the HLEQ. To determine disability levels, we used the physical component summary score (PCS) of the Medical Outcomes Study 36-Item Short Form (SF-36), a widely-used, validated self-

For peer review only

assessment tool. A score of 100 represents no disability and 0 represents a high level of disability.[25] PCS scores were dichotomized above and below the median.

Assessment of area deprivation – exposure

To examine area deprivation, we used the Townsend Index.[26,27] This index is a composite measure of four variables obtained from the 1991 Census: 1) percentage of economically active residents over age 16 who are unemployed, 2) percentage of households that do not possess a car, 3) percentage of private households that are not owner occupied, and 4) percentage of private households that are overcrowded (have more than 1 person per room). These variables were obtained at the level of the enumeration district. For each variable, Z scores were obtained by dividing the mean by the standard deviation (across enumeration districts in England). The Z-values of the four variables were added together to produce a Townsend index score. Positive values of the index indicate areas that are more deprived, while negative values indicate areas that are less deprived; 0 represents the national mean. The postal codes of participants were record linked to enumeration districts, and participants were considered to live in deprived areas depending on the Townsend index score assigned to their enumeration district.

For peer review only

Ascertainment of sense of coherence

The HLEQ included a three-item SOC questionnaire[28] that assessed each of the SOC constructs. The following questions were used to assess each construct:

Comprehensibility:

Do you usually feel that the things that happen to you in your daily life are hard to understand?

Manageability:

Do you usually see a solution to problems and difficulties that other people find hopeless?

Meaningfulness:

Do you usually feel that your daily life is a source of personal satisfaction?

Participants were given the choice of responding to these questions with yes, usually; yes, sometimes; and no. Comprehensibility was reverse scored, and all items were then summed to provide a total SOC scale ranging from 0 to 6. Higher scores represent weaker SOC.

For peer review only

Statistical analysis

Characteristics of the participants were compared by GAD status. We used correlated data analysis to assess the association between individual- and area-level risk factors of GAD in women and men, separately. A population-average model was constructed, which accounted for the potential correlation introduced by the clustering of individuals within enumeration districts. To estimate the population-average effect of the risk factors of interest on past-year GAD, we used generalized estimating equations. As past-year GAD represents a binary outcome (yes/no) and the intra-cluster correlation is assumed to be equal, GEE with a logit link and an exchangeable correlation structure was used. Adjusted odds ratios (OR) and 95% confidence intervals based on robust standard errors were estimated. Since the number of observations per cluster for those with low and high SOC was too small, standard multivariate logistic regression was conducted and results of this paper are based on the latter.

Individual-level measures consisted of demographic and socio-economic status variables, whereas the area-level measure was the Townsend index. Townsend index scores were used to create a dichotomous variable, with 0 as the cut-point (representing the national average).

SOC was split at the median (of 2) and participants below this cut-point were classified as weak on SOC, while those above this cut-point had a strong SOC. The interaction between SOC and GAD in women was assessed. After this, analyses were conducted separately for those with strong and weak levels of SOC. First, unadjusted effect estimates were determined. Next, models were constructed that adjusted for 1) age, social class,

For peer review only

educational attainment; then for 2) age, social class, educational attainment, lifetime history of MDD; and finally for 3) age, social class, educational attainment, lifetime history of MDD, physical diseases, and disability level. Age was assessed as a categorical variable. Models were constructed for participants with complete measurements on all covariates. The brackets show the reference categories that were used for each categorical variable when it was entered in the models - deprivation: no [ref] vs. yes; GAD: no [ref] vs. yes; education: high [ref] vs. low; marital status: married [ref] vs. not married; social class: non-manual [ref] vs. manual; lifetime MDD: no [ref] vs. yes; prevalent physical disease: no [ref] vs. yes; disability level: low [ref] vs. high. These reference categories were based on the literature. Choosing other groupings for the potential confounders would not have changed the results. It was not possible to group the GAD variable otherwise, and area deprivation was analysed in accordance with the literature.

To arrive at the study size, we went through the following steps: of the 30,445 who completed the baseline HLQ, we retained those participants (both men and women) who completed the HLEQ (20,921), and of these, we kept only women with complete data on all covariates (10,277). (Figure 1)

Patient involvement

There were no patients involved in the development of the research question and outcome measures, the design of the study, or the recruitment to and conduct of the study.

For peer review only

Results

77,630 people from general practices in Norfolk were invited to take part in the study, and of these, 30,445 consented.[23] The characteristics of responders versus non-responders are compared in Appendix 1; compared to non-responders, those who took part consisted of slightly more women and slightly younger participants. Of the 30,445 people recruited at baseline, 20,921 completed the HLEQ during follow-up.[23,24] Of those who completed the HLEQ, 10,277 women were retained for analysis in this study, because they had complete data on all covariates. The number of missing observations for each covariate was: 1 for age, 7 for education, 23 for marital status, 303 for social class, 35 for employment, 46 for Townsend index, 883 for disability, 321 for MDD, and 300 for GAD. Participants were assessed between 1993 and 2000 (followed for 7 years) (Figure 1).

In 1996-99, GAD was present in 261 out of 10,277 (2.5%) women. Table 1 shows sociodemographic, medical history, and lifestyle characteristics for women with a weak and strong SOC.

For peer review only

Table 1: Distribution of characteristics for women (n=10,277) with weak and strong SOC who completed the HLEQ questionnaire in the EPIC-Norfolk cohort

	Weak SOC		Strong SOC	
**Characteristic**	**Number with characteristic**	**Percentage and number with past-year GAD**	**Number with characteristic**	**Percentage and number with past-year GAD**
**Socio-demographics**				
**Age (years)**				
<65	1997	6.9 (137)	4768	1.6 (78)
>=65	996	2.7 (27)	2516	0.8 (19)
**Education**[‡]				
Low	1359	4.6 (62)	2668	0.8 (21)
High	1634	6.2 (102)	4616	1.7 (76)
**Marital status**				
Married	2062	5.5 (114)	5651	1.2 (69)
Not married[*]	931	5.4 (50)	1633	1.7 (28)
**Social class**[¥]				
Manual	1262	4.9 (62)	2555	1.1 (27)
Non-manual	1731	5.9 (102)	4729	1.5 (70)
**Employed**				
Yes	1180	5.7 (67)	2875	1.4 (40)
No	1813	5.4 (97)	4409	1.3 (57)
**Townsend index**				
**Deprivation**				
Yes (>0)	534	8.4 (45)	1103	1.7 (19)
No (<=0)	2459	4.8 (119)	6181	1.3 (78)
**Health status**				
**Prevalent physical disease**[+]				
Yes	1684	6.2 (104)	3976	1.8 (70)
No	1309	4.6 (60)	3308	0.8 (27)
**Disability level**				
High[¶]	1718	6.3 (108)	3544	1.8 (64)
Low	1275	4.4 (56)	3740	0.9 (33)
**Lifetime MDD**				
Yes	738	14.0 (103)	1190	5.4 (64)
No	2255	2.7 (61)	6094	0.5 (33)

[+] Prevalent physical disease: respiratory disease (asthma and bronchitis), allergies (allergies and hay fever), stroke, heart attack, cancer, diabetes, thyroid conditions, arthritis

[¥] Manual: skilled manual, semi-skilled, non-skilled; non-manual: professionals, managerial, skilled non-manual

[‡] High education: O-level, A-level, degree; low education: refers to no education

[*] Other: divorced, separated, widowed

[¶] Below the median PCS value of 50.6

For peer review only

Among women with a weak SOC, those who also had GAD were more likely to be younger, have high educational attainment, live in areas of high deprivation, and have had pre-existing health conditions or show prevalent physical disease, high disability, and lifetime MDD. In the group with strong SOC, similar patterns were found as for those with anxiety.

During the 6-year follow-up period, there were a total of 261 GAD cases in women. A weak SOC was found in 2993 women, while a strong SOC was present in 7284 women. When the interaction between SOC and GAD was assessed, the p-value was 0.221. Tables 2 and 3 show the unadjusted (Model A) and adjusted odds ratios (Models B-E) associated with GAD in those with a weak and strong SOC, respectively.

For peer review only

Table 2: Odds ratios for women with a weak SOC who completed the HLEQ questionnaire in 1996-00 (women with weak SOC sample size=2993)

	Odds ratios (OR) and 95% CI				
**Characteristic**	**Unadjusted**	**Model A[1]**	**Model B[2]**	**Model C[3]**	**P-value for Model C**
**Socio-demographics**					
**Age**					
<65	2.64 (1.74, 4.02)	3.34 (2.11, 5.30)	2.48 (1.54, 3.97)	2.67 (1.66, 4.29)	<0.0001
>=65	1.00	1.00	1.00	1.00	
**Education[‡]**					
Low	0.72 (0.52, 0.99)	0.76 (0.54, 1.08)	0.82 (0.58, 1.18)	0.82 (0.57, 1.18)	0.288
High	1.00	1.00	1.00	1.00	
**Marital status**					
Married	1.00	1.00	1.00	1.00	
Not married[*]	0.97 (0.69, 1.37)	1.05 (0.73, 1.50)	0.85 (0.59, 1.23)	0.84 (0.58, 1.22)	0.362
**Social class[¥]**					
Manual	0.83 (0.60, 1.14)	0.81 (0.57, 1.14)	0.83 (0.58, 1.17)	0.80 (0.56, 1.14)	0.216
Non-manual	1.00	1.00	1.00	1.00	
**Employed**					
Yes	1.00	1.00	1.00	1.00	
No	0.94 (0.68, 1.29)	1.53 (1.08, 2.16)	1.40 (0.98, 2.01)	1.31 (0.91, 1.88)	0.145
**Townsend index**					
**Deprivation**					
Yes (>0)	1.81 (1.27, 2.59)	2.04 (1.41, 2.96)	1.97 (1.34, 2.88)	1.99 (1.35, 2.92)	0.001
No (<=0)	1.00	1.00	1.00	1.00	
**Health status**					
**Lifetime MDD**					
Yes	5.83 (4.20, 8.11)		5.26 (3.76, 7.35)	5.11 (3.65, 7.16)	<0.0001
No	1.00		1.00	1.00	
**Prevalent physical disease[+]**					
Yes	1.37 (0.99, 1.90)			1.21 (0.85, 1.71)	0.292
No	1.00			1.00	
**Disability level**					
High[¶]	1.46 (1.05, 2.03)			1.51 (1.06, 2.16)	0.023
Low	1.00			1.00	

1. Adjusted for age, SES (education, marital status, social class)
2. Adjusted for age, SES, lifetime MDD
3. Adjusted for age, SES, lifetime MDD, physical disease and disability

[+] Prevalent physical disease: respiratory disease (asthma, bronchitis), allergies (allergies, hay fever), stroke, heart attack, cancer, diabetes, thyroid conditions, arthritis

[¥] Manual: skilled manual, semi-skilled, non-skilled; non-manual: professionals, managerial, skilled non-manual

[‡] High education: O-level, A-level, degree; low education: refers to no education

[*] Other: divorced, separated, widowed

[¶] Below the median PCS value of 50.6

For peer review only

Table 3: Odds ratios for women with a strong SOC who completed the HLEQ questionnaire in 1996-00 (women with a strong SOC sample size =7284)

	Odds ratios (OR) and 95% CI				
**Characteristic**	**Unadjusted**	**Model A[1]**	**Model B[2]**	**Model C[3]**	**P-value for Model C**
**Socio-demographics**					
**Age**					
<65	2.19 (1.32, 3.62)	2.61 (1.50, 4.56)	1.93 (1.09, 3.41)	2.17 (1.22, 3.86)	0.009
>=65	1.00	1.00	1.00	1.00	
**Education[‡]**					
Low	0.47 (0.29, 0.77)	0.54 (0.32, 0.90)	0.59 (0.35, 0.99)	0.59 (0.35, 1.00)	0.049
High	1.00	1.00	1.00	1.00	
**Marital status**					
Married	1.00	1.00	1.00	1.00	
Not married	1.41 (0.91, 2.20)	1.56 (0.99, 2.46)	1.24 (0.78, 1.98)	1.21 (0.76, 1.94)	0.429
**Social class[¥]**					
Manual	0.71 (0.46, 1.11)	0.84 (0.52, 1.33)	0.86 (0.53, 1.38)	0.83 (0.51, 1.34)	0.440
Non-manual	1.00	1.00	1.00	1.00	
**Employed**					
Yes	1.00	1.00	1.00	1.00	
No	0.93 (0.62, 1.40)	1.46 (0.94, 2.27)	1.44 (0.92, 2.26)	1.25 (0.79, 1.97)	0.348
**Townsend index**					
**Deprivation**					
Yes (>0)	1.37 (0.83, 2.27)	1.43 (0.86, 2.38)	1.31 (0.78, 2.21)	1.28 (0.76, 2.16)	0.351
No (<=0)	1.00	1.00	1.00	1.00	
**Health status**					
**Life-time MDD**					
Yes	10.44 (6.83, 15.96)		9.35 (6.08, 14.39)	8.62 (5.59, 13.29)	<0.0001
No	1.00		1.00	1.00	
**Prevalent physical disease[+]**					
Yes	2.18 (1.39, 3.40)			1.73 (1.09, 2.74)	0.020
No	1.00			1.00	
**Disability level**					
High[¶]	2.07 (1.35, 3.15)			1.92 (1.23, 3.00)	0.004
Low	1.00			1.00	

For peer review only - http://bmjopen.bmj.com/site/about/guidelines.xhtml

1. Adjusted for age, SES (education, marital status, social class)
2. Adjusted for age, SES, lifetime MDD
3. Adjusted for age, SES, lifetime MDD, physical diseases and disability

[+] Prevalent physical disease: respiratory disease (asthma, bronchitis), allergies (allergies, hay fever), stroke, heart attack, cancer, diabetes, thyroid conditions, arthritis

[¥] Manual: skilled manual, semi-skilled, non-skilled; non-manual: professionals, managerial, skilled non-manual

[‡] High education: O-level, A-level, degree; low education: refers to no education

[*] Other: divorced, separated, widowed

[¶] Below the median PCS value of 50.6

For peer review only

Analyses that adjusted for age, education, marital status, social class, and employment status showed that area deprivation was significantly associated with increased risk for GAD in women with a weak SOC (OR=1.93, 95% CI: 1.34, 2.78) (table 2), but area deprivation was not significantly associated with anxiety in those with strong SOC (OR=1.42, 95% CI: 0.86, 2.37) (table 3). In women with a weak SOC (table 2), further adjustment for lifetime MDD slightly attenuated the effect estimate, though the association between area deprivation and anxiety remained highly significant (OR=1.86, 95%: 1.27, 2.74). When prevalent physical disease and disability level were added to the final model, the effect estimate remained almost unchanged compared to the previous model; among women with poor coping skills, those living in deprived areas had an 88% higher likelihood of having anxiety than women living in less deprived areas (OR=1.88, 95% CI: 1.28, 2.77). For women with a strong SOC (table 3), area deprivation was associated with a small increased risk of having GAD in progressively adjusted models; however, none of the effect estimates reached statistical significance. In the fully-adjusted model, women with a strong SOC and living in deprivation had a 22% higher chance of having GAD compared to women living in less deprived areas, but this did not reach statistical significance (OR=1.28, 95% CI: 0.76, 2.16).

We carried out multiple imputations for missing data (Appendix 2); the effect estimate became even stronger for women with a weak SOC and living in deprivation (OR=2.28, 95% CI: 1.61, 3.23), and the association between deprivation and anxiety become even weaker for women with a strong SOC (OR=1.13, 95% CI: 0.68, 1.90).

For peer review only

Discussion

In this large, population-based study, we found that area deprivation significantly increased the risk for generalized anxiety disorder (GAD) in women, but particularly in those with poor coping skills. Coping skills or sense of coherence (SOC) appeared to mediate the association between area deprivation and anxiety. Women living in deprivation and with poor coping or a weak SOC were at a particularly high risk for having anxiety after controlling for important confounders. Although women with a strong SOC showed a slight increased risk of anxiety if living in disadvantaged circumstances, the association between area deprivation and GAD was statistically non-significant in women who were able to cope well and the effect estimate was much smaller than that of the former group (women with poor coping). A statistically significant association between area deprivation and GAD persisted in women with a weak SOC after adjustment for age, marital status, education level, social class, major depressive disorder, chronic physical diseases, and disability. In contrast, having a strong SOC seemed to be protective for women living in deprived areas. Having a strong SOC rendered the association between area deprivation and anxiety statistically non-significant.

Deprived areas are often associated with low social integration and poor social control. Emile Durkheim showed that low social integration can lead to a sense of meaninglessness among individuals, and this can give rise to poor mental health and suicide.[29] SOC is a way of viewing life as meaningful and comprehensible, and our study shows that SOC can moderate the association between area deprivation and GAD in women.

For peer review only

Strengths and limitations of this study, and future research

This is the largest, population-based study of the association between area deprivation and GAD in women, and to determine whether coping resources or SOC mediates the association between area deprivation and anxiety. We had access to a large sample of over 10,000 women living in the community. We used a clinically relevant measure of anxiety, and GAD was defined according to the Diagnostic and Statistical Manual of Mental Disorders, fourth version (DSM-IV). Although GAD affects a substantial number of people, even more experience subthreshold cases of anxiety disorders. Subthreshold cases have also been associated with impairment and disability; therefore, future research should assess associations with subclinical anxiety. Finally, the measure of SOC that we used in this study has been reported to be valid, reliable, and theoretically-sound [30].

We used detailed health and lifestyle questionnaires to extract information on demographics, social class, and major chronic physical diseases, and controlled for these factors in our analyses. We used a validated and reliable measure of disability, which we adjusted for in our models. We had a large list of self-reported physician diagnoses that we used to establish medical histories for participants, though three issues might arise with this approach. First, the residual effect of diseases not captured by our study but that are associated with area deprivation and anxiety, may be present. Second, medical diagnoses were not verified by clinicians, leading to possible misclassification. Third, past illness may have been under-reported, leading to misclassification bias and attenuation of effect estimates. We may have overadjusted our models with the inclusion of disability, because this might be part of the expression of psychiatric illness. This may have reduced effect estimates. Our objective was to assess the links between deprivation, SOC, and anxiety in

For peer review only

women. Although it was out of scope for the present study, we were unable to examine the same objectives in men: there were very few men with a strong SOC living in deprivation and with GAD. Therefore, analyses in this sub-group would not have been robust. Future studies should undertake this assessment.

At baseline, people who consented to take part in EPIC-Norfolk agreed to fill out detailed health and lifestyle questionnaires over the duration of the study period; therefore, healthy volunteer effect may have biased our findings. Participants in EPIC-Norfolk tend to be somewhat healthier and more affluent than the general population, therefore, results from this study cannot be generalized to extremely deprived areas. If the most deprived areas would have been included, we would expect the association between area deprivation and anxiety to be even stronger in women with a weak SOC. Also, when comparing the demographic characteristics of responders versus non-responders (Appendix 1), we found that participants were slightly younger and slightly more women than men consented.

Also, it may be that participants with poorer mental health may have moved to more deprived neighbourhoods; however, reverse causality seems unlikely as an explanation for our findings. Also, deprivation was measured before anxiety in this study; however, SOC was examined at the same time point as GAD, rendering this study cross-sectional.

Non-participation in our study may have contributed to non-differential misclassification and attenuation of effect estimates. Although our study is observational in nature and cannot confirm causality between area deprivation and generalized anxiety disorder in women with a strong and weak SOC, a rigorous analysis based on observational data is a

For peer review only

reasonable way of examining this relationship. When we conducted multiple imputations, the effect estimate for women with a weak SOC became even greater, and among women with a strong SOC, it attenuated towards the null. Our study provides a valuable step forward and is the first to shed light on the importance of coping in people with GAD living in disadvantaged circumstances.

Comparison with other studies

This is the largest, population-based study to consider the association between area deprivation and GAD in women, and to determine whether SOC mediates this association. Most of the literature on coping and sense of coherence is limited. Most studies have small sample sizes, and measure people's coping abilities in relation to feelings of stress, history of stressful life events, or exposure to stressful circumstances, such as, wars. There is a paucity of research examining the living context, such as, area deprivation, and no studies have assessed whether the link between area-level circumstances and anxiety disorders can be mediated by coping mechanisms. The literature on coping uses highly select samples; therefore, results cannot be generalized to the larger population. Also, incomplete adjustment of covariates makes it difficult to determine whether findings from these studies are not better explained by the residual effect of other factors that have not been accounted for, such as, lifestyle and personal socio-economic circumstances. Across studies, there is large heterogeneity in the definitions used to define coping, with many focusing on factors, such as, hardiness, optimism, and negative emotions, rather than SOC. In sum, it is difficult to understand the links between the living context, coping abilities, and mental health from the literature; however, the studies that have been conducted are a good starting point.

For peer review only

A UK study of over 3000 people [31] showed that SOC was linked to self-rated health; however, the moderating effect of coping was not assessed. Research[32] on people living in Negev communities in Israel showed that those exposed to trauma and severe stress-provoking situations, but who had a strong SOC, were least likely to develop stress.[32] In a study of French adults [28], SOC buffered the effect of adversity on psychological well-being. In another study of Holocaust survivors [21], SOC moderated the association between early childhood deprivation and posttraumatic stress in old age. Both of these latter studies, however, were small, failed to adjust for important confounders, such as sociodemographic factors and disability, and did not examine individual psychiatric disorders diagnosed according to valid and reliable criteria, such as, the DSM. In the study on child Holocaust survivors [21], exposure to trauma was measured in early life, while posttraumatic stress in old age. Since participants were required to report traumas experienced in childhood, this might have led to recall bias. Our study expands on previous research and is the first to investigate the moderating effect of coping skills (sense of coherence [SOC]) on the risk of developing generalized anxiety disorder (GAD) in women living in deprived circumstances.

Mechanism of effect

Living in a derived area can increase anxiety in women because of biological and social factors.[16] The stress of living in deprivation can increase the risk for inflammation and HPA-axis dysregulation, which can lead to GAD.[34, 35, 36] This, combined with the multiple roles that women are increasingly taking on (income earner, child-bearer, and carer of elderly relatives) [37], means that coping is particularly relevant for women living in disadvantaged circumstances. A strong SOC is linked to high quality of life, and good

For peer review only

physical and mental health [18,19]. Our study shows that SOC can buffer the effect of area deprivation on risk of anxiety.

Implications

The absolute number of people living in deprived conditions is large worldwide, and significant numbers will have been affected by generalized anxiety disorder (GAD).[37] For the first time, we show that sense of coherence (SOC) moderates the association between area deprivation and anxiety in women. Future research should replicate our analysis using larger samples and determine the specific components of SOC that attenuate the effect of deprivation on mental health. Interventions can then be developed to target components of SOC to increase people's coping resources. Treatment for generalized anxiety disorder exists, with psychotherapy and pharmacotherapy being commonly prescribed. However, success rates are fairly low, patients relapse, and some fail to experience any symptom improvement. Costs to the health care system related to anxiety are substantial. Therefore, targeting people's coping resources could represent another option for people with anxiety, including those who do not experience symptom improvement following commonly-prescribed therapies. Targeting SOC could also represent a better option for people who have faced extreme circumstances and adversity, and who may have difficultly dealing with the traumas directly as during psychotherapy. Interventions should take these findings into account, and mental health policy should also consider improving living environments to decrease the burden of anxiety in women.

For peer review only

489 Acknowledgements: OR received a PhD studentship from the National Institute for Health Research.

Competing interest: All authors have completed the ICMJE uniform disclosure form at www.icmje.org/coi_disclosure.pdf and declare: no support from any organisation for the submitted work; no financial relationships with any organizations that might have an interest in the submitted work in the previous three years; no other relationships or activities that could appear to have influenced the submitted work.

Funding: This work was supported by the Medical Research Council UK (grant number SP2024-0201 and SP2024-0204) and Cancer Research UK (grant number G9502233).

Author contributions: OR (corresponding author) had the idea for and conducted the analysis, and wrote the article. CB critically reviewed drafts of the manuscript, KK edited versions of the paper; PS and NW provided feedback into the analysis. OR, CB, KK, LL, PS, and NW contributed to the interpretation of data for the work, agreed to be accountable for all aspects of the work, gave final approval of the version to be published, and made substantial contributions to the analysis and interpretation of data. OR, CB, KK, LL, PS, and NW have seen and approved the final version. OR, CB, KK, LL, PS, and NW had full access to all the data in the study and take responsibility for the integrity of the data and the accuracy of the data analysis. OR acts as guarantor of the study.

For peer review only

Transparency declaration: OR affirms that the manuscript is an honest, accurate, and transparent account of the study being reported; that no important aspects of the study have been omitted; and that any discrepancies from the study as planned have been explained.

Role of study sponsors and statement of independence: The funding sources had no role in the design and conduct of the study; collection, management, analysis, and interpretation of the data; and preparation, review, or approval of the manuscript.

Ethical approval: The study has ethics committee approval from Norfolk Ethics Committee (Rec Ref: 98CN01) and all participants gave informed consent.

Data sharing: No additional data available. Original dataset requests should be sent to the corresponding author. Please contact O Remes at or260@medschl.cam.ac.uk for questions about the statistical code.

Copyright: "The Corresponding Author has the right to grant on behalf of all authors and does grant on behalf of all authors, a worldwide licence to the Publishers and its licensees in perpetuity, in all forms, formats and media (whether known now or created in the future), to i) publish, reproduce, distribute, display and store the Contribution, ii) translate the Contribution into other languages, create adaptations, reprints, include within collections and create summaries, extracts and/or, abstracts of the Contribution, iii) create any other

For peer review only

derivative work(s) based on the Contribution, iv) to exploit all subsidiary rights in the Contribution, v) the inclusion of electronic links from the Contribution to third party material where-ever it may be located; and, vi) licence any third party to do any or all of the above."

For peer review only

References

1. American Psychiatric Association. (1994). *Diagnostic and Statistical Manual of Mental Disorders* (4th edn). Washington, DC: APA.
2. Kessler R, Keller M, Wittchen H. The epidemiology of generalized anxiety disorder. *Psychiatr Clin North Am* 2001;24:19–39, doi: http://dx.doi.org/10.1016/S0193-953X(05)70204-5 [published Online First 29 June 2005].
3. Stein MB, Sareen J. Generalized anxiety disorder. *New Engl J Med* 2015;373:2059–2068, doi: 10.1056/NEJMcp1502514 [published Online First 19 November 2015].
4. Hoge E, Ivkovic A, Fricchione G. Generalized anxiety disorder: diagnosis and treatment. *BMJ* 2012;345,e7500, doi: http://dx.doi.org/10.1136/bmj.e7500 [published Online First 27 November 2012].
5. Nepon J, Belik S, Bolton J, Sareen J. The relationship between anxiety disorders and suicide attempts: findings from the National Epidemiologic Survey on Alcohol and Related Conditions. *Depress Anx* 2010;27:791–798, doi: 10.1002/da.20674 [published Online First 9 March 2010].
6. Revicki DA, Travers K, Wyrwich KW. Humanistic and economic burden of generalized anxiety disorder in North America and Europe. *J Affect Disord* 2012;140:103–12.
7. Simpson HB, Neria Y, Lewis-Fernandez R, Schneier F. Anxiety disorders: theory, research and clinical perspectives. Cambridge University Press, 2010.
8. Grant BF, Hasin DS, Stinson FS, et al. Prevalence, correlates, co-morbidity, and comparative disability of DSM-IV generalized anxiety disorder in the USA: results from the National Epidemiologic Survey on Alcohol and Related Conditions. *Psychol Med* 2005;35:1747, doi: 10.1017/S0033291705006069 [published Online First 5 October 2005].

For peer review only

9. The NHS Information Centre for health and social care, Leeds. Adult psychiatric morbidity in England, 2007 Results of a household survey. http://www.esds.ac.uk/doc/6379/mrdoc/pdf/6379research_report.pdf (accessed 29 July 2014).

10. Fryers T, Melzer D, Jenkins R. Social inequalities and the common mental disorders: a systematic review of the evidence. *Soc Psychiatry Psychiatr Epidemiol* 2003;38:229–237, doi: 10.1007/s00127-003-0627-2.

11. Zhang X, Norton J, Carriere I, Ritchie K, Chaudieu I, Ancelin ML. Risk factors for late-onset generalized anxiety disorder: results from a 12-year prospective cohort (the ESPRIT study). *Transl Psychiatry* 2015;5:e536, doi: 10.1038/tp.2015.31 [published Online First 31 March 2015].

12. Subramanian S, Kawachi I. Income inequality and health: what have we learned so far?. *Epidemiol Rev* 2004;26:78–91, doi: 10.1093/epirev/mxh003.

13. Pickett KE, Wilkinson RG. Income inequality and health: a causal review. *Soc Sci Med* 2014;128:316–326, doi: 10.1016/j.socscimed.2014.12.031 [published Online First 30 December 2014].

14. Pattyn E, Van Praag L, Verhaeghe M, Levecque K, Bracke P. The association between residential area characteristics and mental health outcomes among men and women in Belgium. *Arch Public Health* 2011;69:3, doi: 10.1186/0778-7367-69-3 [published Online First 24 October 2011].

15. Stafford M, Cummins S, Macintyre S, Ellaway A, Marmot M. Gender differences in the associations between health and neighbourhood environment. *Soc Sci Med* 2005;60:1681–1692, doi: 10.1016/j.socscimed.2004.08.028 [published Online First 5 November 2004].

For peer review only

16. Remes O, Wainwright N, Surtees P, Lafortune L, Khaw K, Brayne C. Sex differences in the association between area deprivation and generalized anxiety disorder: British population study. *BMJ Open* 2017 (in press).

17. Gender differences in the association between common mental disorders and regional deprivation in Ireland. *Prof Geogr* 2015;68:129-137 doi: 10.1080/00330124.2015.1054020 [published Online First 24 July 2015].

18. Eriksson M, Lindstrom B. Antonovsky's sense of coherence scale and its relation with quality of life: a systematic review. *J Epidemiol Community Health* 2007;61:938-944, doi: 10.1136/jech.2006.056028.

19. Eriksson M, Lindstrom B. Antonovsky's sense of coherence scale and the relation with health: a systematic review. *J Epidemiol Community Health* 2006;60:376-381, doi: 10.1136/jech.2005.041616.

20. Wainwright NWJ, Surtees PG, Welch AA, Luben RN, Khaw K-T, Bingham SA. Sense of coherence, lifestyle choices and mortality. J Epidemiol Community Health 2008;62:829-831 doi: http://dx.doi.org/10.1136/jech.2007.066464.

21. van der Hal-van Raalte EAM, van IJzendoorn MH, Bakermans-Kranenburg MJ. Sense of coherence moderates late effects of early childhood holocaust exposure. J Clin Psychol 2008;64:1352-67. doi: 10.1002/jclp.20528. [published Online First 24 October 2008].

22. Hayat SA, Luben R, Keevil VL, et al. Cohort profile: a prospective cohort study of objective physical and cognitive capability and visual health in an ageing population of men and women in Norfolk (EPIC-Norfolk 3). *Int J Epidemiol* 2013;1-10 [published Online First 14 June 2013].

23. Surtees P, Wainwright N, Khaw K, Day N. Functional health status, chronic medical

For peer review only

conditions and disorders of mood. *Br J Psychiatry* 2003;183:299–303, doi: 10.1192/bjp.183.4.299 [published Online First 30 September 2003].

24. Ware JE, Snow K, Kosinski M, Gandek B. New England Medical Center Hospital. Health Institute. SF-36 Health Survey: Manual and Interpretation Guide. The Health Institute; Boston, MA: New England Medical Center 1993.

25. National Centre for Research Methods. Townsend deprivation index. http://www.restore.ac.uk/geo-refer/36229dtuks00y19810000.php (accessed 15 April 2014).

26. Shohaimi S, Luben R, Wareham N, et al. Residential area deprivation predicts smoking habit independently of individual educational level and occupational social class. A cross sectional study in the Norfolk cohort of the European Investigation into Cancer (EPIC-Norfolk). *J Epidemiol Community Health* 2003;57:270–276, doi: 10.1136/jech.57.4.270.

27. Surtees PG, Wainwright NWJ, Luben R, Khaw K-T, Day NE. Mastery, sense of coherence, and mortality: evidence of independent associations from the EPIC-Norfolk prospective cohort study. *Health Psychol* 2006;25:102-10, doi: 10.1037/0278-6133.25.1.102.

28. Durkheim E. Suicide – a study in sociology. Simon and Schuster 2010.

29. Eriksson M, Lindstrom B. Validity of Antonovsky's sense of coherence scale: a systematic review. *J Epidemiol Community Health* 2005;59:460-6. doi: http://dx.doi.org/10.1136/jech.2003.018085.

30. Walsh D, McCartney G, McCullough S, Buchanan D, Jones R. Comparing Antonovsky's sense of coherence scale across three UK post-industrial cities. BMJ Open 2014;4:e005792, doi: http://dx.doi.org/10.1136/bmjopen-2014-005792.

31. Braun-Lewensohn O, Sagy S. Community resilience and sense of coherence as

For peer review only

protective factors in explaining stress reactions: comparing cities and rural communities during missiles attacks. *Community Mental Health J* 2014;50:229-234, doi: 10.1007/s10597-013-9623-5 [published Online First 9 June 2013].

32. Gana K. Is sense of coherence a mediator between adversity and psychological wellbeing in adults? *Stress and Health* 2001;17:77-83, doi: 10.1002/smi.882 [published Online First 26 February 2001].

33. Pitsavos C, Panagiotakos DB, Papageorgiou C, Tsetsekou E, Soldatos C, Stefanadis C. Anxiety in relation to inflammation and coagulation markers, among healthy adults: the ATTICA study. *Atherosclerosis* 2006;185:320–6. doi: 10.1016/j.atherosclerosis.2005.06.001. [published Online First 11 July 2005].

34. Hoehn-Saric R, McLeod D, Funderburk F, Kowalski P. Somatic symptoms and physiologic responses in generalized anxiety disorder and panic disorder. *Arch Gen Psychiatry* 2004;61:913, doi: 10.1001/archpsyc.61.9.913.

35. Chaudieu I, Beluche I, Norton J, Boulenger JP, Ritchie K, Angelin ML. Abnormal reactions to environmental stress in elderly persons with anxiety disorders: evidence from a population study of diurnal cortisol changes. *J Affect Disord* 2008;106:307–313, doi: 10.1016/j.jad.2007.07.025 [published Online First 28 August 2007].

36. Remes O, L Lafortune, K-T Khaw, C Brayne. Sex and gender in psychiatry. Lancet Psychiatry 2017;4:e1, doi: http://dx.doi.org/10.1016/S2215-0366(16)30445-X.

37. Whiteford HA, Degenhardt L, Rehm J, et al. Global burden of disease attributable to mental and substance use disorders: findings from the Global Burden of Disease Study 2010. *Lancet* 2013;9904:1575-1586 doi: http://dx.doi.org/10.1016/S0140-6736(13)61611-6 [published Online First 29 August 2013].

For peer review only

659 **Appendix 1: Characteristics of participants who consented (n=30,445) and refused**

660 **(n=43,452) to take part in the EPIC-Norfolk cohort study**

	**Percentage (number)**	
**Characteristic**	**Consented**	**Did not consent**
**Age**		
<50	27.5 (8366)	33.7 (14647)
50-60	30.3 (9230)	29.5 (12819)
60-70	32.5 (9879)	27.4 (11898)
>=70	9.8 (2970)	9.4 (4088)
**Sex**		
Female	55.0 (16744)	49.0 (21296)
Male	45.0 (13701)	51.0 (22156)

661

662

For peer review only

Appendix 2 – Supplementary Material

We imputed missing data separately for women with a weak and strong sense of coherence (SOC). Based on the literature, we identified 12 potential auxiliary variables; however, we retained eight variables that were correlated with the variables in our model and were good predictors of the missing status (based on statistical tests). Our imputation model included all variables in the analysis model and the auxiliary variables.

To retain as much information as possible, we conducted the imputations on non-transformed data-the original variables in our dataset. We imputed data using the fully conditional specification, and specified a linear regression model for continuous data that were normally distributed; predictive mean matching for continuous data that were not normally distributed; and logistic regression for categorical variables. Variable estimates were subsequently averaged from 100 imputed datasets using Rubin's rules (we transformed the data before running the analytic model of interest within each of the imputed datasets).[1]

We checked whether the imputations were acceptable by comparing 1) the means, standard deviations, and plots of recorded and imputed values for continuous variables, and 2) the frequencies and percentages of recorded and imputed values for each level of categorical variables.

Analyses were done using SAS 9.3 and p-values less than 0·05 were considered statistically significant.

Auxiliary variables used in the imputation model

Variable	Questionnaire	Description of variable
**Psychological factors**		
Paternal affection	Health and Life Experiences Questionnaire (HLEQ)	Self-reported paternal affection using the Rossi scale[2]. The scale assessed items, such as, family composition, parental divorce and death, quality of relationship with the father and amount of affection received.
Mastery	Health and Life Experiences Questionnaire (HLEQ)	Self-reported using the Pearlin and Schooler Mastery Scale. Mastery is having a sense of control over one's life or the belief that one has control over future important life circumstances. It represents a coping resource that people use to manage or attenuate the impact of stressors, and this in turn, has an influence on health and health behaviours.[3,4]
Neuroticism	Health and Life Experiences Questionnaire (HLEQ)	Self-reported using the Eysenck Personality Inventory. A tendency towards experiencing negative, distressing emotions.[5]
**Sociodemographic factors**		
School age	Health and Lifestyle (HLQ) Questionnaire	Self-reported age when participant left school.

For peer review only

Physical health		
Systolic blood pressure	Baseline health check	Systolic blood pressure measured using an Accutorr noninvasive oscillometric blood pressure monitor; mean of two measurements in mmHg
Diastolic blood pressure	Baseline health check	Diastolic blood pressure measured using an Accutorr noninvasive oscillometric blood pressure monitor; mean of two measurements in mmHg
History of high cholesterol	Health and Lifestyle Questionnaire (HLQ)	Self-reported history of high cholesterol
History of psychiatric illness	Health and Lifestyle Questionnaire (HLQ)	Self-reported history of other psychiatric illness

The questionnaires used for these variables have been previously described in the methods.

The following auxiliary variables were not included in the imputation model, because they were not correlated with the variables in our model and were not good predictors of the missing status (following tests using Pearson's/Spearman's correlation coefficient and t-tests/chi-square tests): composite measure of maternal affection using the Rossi scale[2], and self-reported history of: migraine, benign tumours, psychiatric illness, and back pain.

References

1. Berglund P, Heeringa S. Multiple imputation of missing data using SAS. Cary, NC: *SAS Institute Inc*, 2014.
2. Rossi AS. Caring and doing for others: social responsibility in the domains of family, work, and community. Chicago: *University of Chicago Press*, 2001.
3. Pearlin LI, Menaghan EG, Lieberman MA, Mullan JT. The stress process. *J Health Soc Behav* 1981;22:337–56.
4. Pearlin LI, Schooler C. The structure of coping. *J Health Soc Behav* 1978;19:2–21.
5. Shipley BA, Weiss A, Der G, Taylor MD, Deary IJ. Neuroticism, extraversion, and mortality in the UK Health and Lifestyle Survey: a 21-year prospective cohort study. *Psychosom Med* 2007;69:923–31 doi: 10.1097/PSY.0b013e31815abf83 [published Online First 8 November 2007].

For peer review only

Figure 1 - Flowchart of EPIC-Norfolk cohort

This is a flowchart showing the number of participants at each study stage: the number approached to participate in the EPIC-Norfolk study, the number enrolled at baseline, and with complete data on all covariates. The EPIC-Norfolk study consists of middle-aged and older British people.

For peer review only

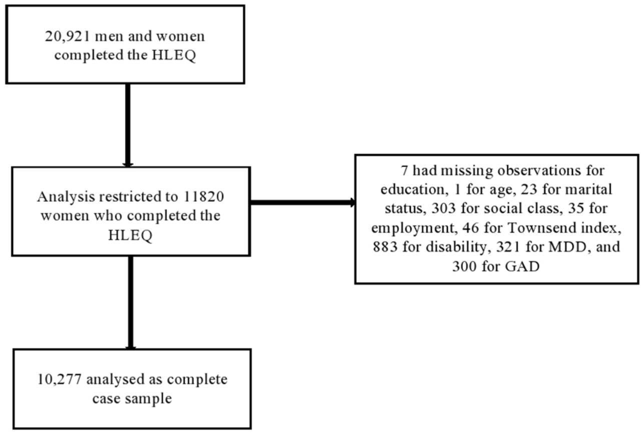

449x582mm (72 x 72 DPI)

Please see the article line numbers (column on the right) and the explanations provided.

STROBE Statement—checklist of items that should be included in reports of observational studies

	Item No	Recommendation	Line numbers within the article
**Title and abstract**	1	(*a*) Indicate the study's design with a commonly used term in the title or the abstract	7, 39
		(*b*) Provide in the abstract an informative and balanced summary of what was done and what was found	30-64
**Introduction**			
Background/rationale	2	Explain the scientific background and rationale for the investigation being reported	87-129
Objectives	3	State specific objectives, including any prespecified hypotheses	129-131
**Methods**			
Study design	4	Present key elements of study design early in the paper	135
Setting	5	Describe the setting, locations, and relevant dates, including periods of recruitment, exposure, follow-up, and data collection	136-140, 144-146, 153-154
Participants	6	(*a*) *Cohort study*—Give the eligibility criteria, and the sources and methods of selection of participants. Describe methods of follow-up<br>*Case-control study*—Give the eligibility criteria, and the sources and methods of case ascertainment and control selection. Give the rationale for the choice of cases and controls<br>*Cross-sectional study*—Give the eligibility criteria, and the sources and methods of selection of participants	148-150, 135-146
		(*b*) *Cohort study*—For matched studies, give matching criteria and number of exposed and unexposed<br>*Case-control study*—For matched studies, give matching criteria and the number of controls per case	
Variables	7	Clearly define all outcomes, exposures, predictors, potential confounders, and effect modifiers. Give diagnostic criteria, if applicable	152-195, 197-212
Data sources/ measurement	8*	For each variable of interest, give sources of data and details of methods of assessment (measurement). Describe comparability of assessment methods if there is	There were three variables of interest in this study: area deprivation, sense of coherence, and generalized

For peer review only - http://bmjopen.bmj.com/site/about/guidelines.xhtml

		more than one group	anxiety disorder (182-195, 197-212, 152-163). The others are potential confounders – in the methods I list them all, indicate how they were assessed and mention that they were collected through the baseline, self-reported postal HLQ questionnaire as well as the HLEQ.
Bias	9	Describe any efforts to address potential sources of bias	238-239
Study size	10	Explain how the study size was arrived at	248-251
Quantitative variables	11	Explain how quantitative variables were handled in the analyses. If applicable, describe which groupings were chosen and why	In the methods, I mention how the variables were derived based on the raw data provided by participants in the questionnaires.<br><br>Dependent variable: 152-163<br>Area-level measure: 182-195<br>Individual-level measures: 165-180<br>Effect modifier: 197-212<br><br>In the text, I mention that the categorization was done in accordance with the literature, which I cite.
Statistical methods	12	(*a*) Describe all statistical methods, including those used to control for confounding	214-246
		(*b*) Describe any methods used to examine subgroups and interactions	I wanted to determine whether sense of coherence (SOC) was an effect modifier in the association between area deprivation and anxiety. I therefore compared the association between deprivation and anxiety in women with both high and low SOC. Before doing this, I examined the interaction between anxiety and SOC. This is described in the methods.
		(*c*) Explain how missing data were addressed	We indicated that this was a complete-case analysis.
		(*d*) *Cohort study*—If applicable, explain how loss to follow-up was addressed<br>*Case-control study*—If applicable, explain how matching of cases and controls was addressed	Loss to follow-up was not a problem in this study. We were able to track down all participants using various means, unless they expressed that they wished

For peer review only - http://bmjopen.bmj.com/site/about/guidelines.xhtml

Cross-sectional study—If applicable, describe analytical methods taking account of sampling strategy	to be removed from the mailing list. We elaborate on this in the manuscript.
(*e*) Describe any sensitivity analyses	Logistic regression replaced GEE, because of statistical considerations. This was discussed in the methods section.

For peer review only

For peer review only - http://bmjopen.bmj.com/site/about/guidelines.xhtml

**Results**			
Participants	13*	(a) Report numbers of individuals at each stage of study—eg numbers potentially eligible, examined for eligibility, confirmed eligible, included in the study, completing follow-up, and analysed	259-260, 262-265
		(b) Give reasons for non-participation at each stage	We do not have the reasons for non-participation, because these data were not collected when the study was initiated in 1993. There is some information comparing responders versus non-responders – Appendix I in our paper.
		(c) Consider use of a flow diagram	Flow diagram included in submission.
Descriptive data	14*	(a) Give characteristics of study participants (eg demographic, clinical, social) and information on exposures and potential confounders	270, 274-282, 283-286; we provided characteristics for those with vs. without GAD, because we felt it was important to show the characteristics of those exposed vs. non-exposed (see also Table 1)
		(b) Indicate number of participants with missing data for each variable of interest	265-268
		(c) *Cohort study*—Summarise follow-up time (eg, average and total amount)	268
Outcome data	15*	*Cohort study*—Report numbers of outcome events or summary measures over time	270, 288
		Case-control study—Report numbers in each exposure category, or summary measures of exposure	
		Cross-sectional study—Report numbers of outcome events or summary measures	
Main results	16	(*a*) Give unadjusted estimates and, if applicable, confounder-adjusted estimates and their precision (eg, 95% confidence interval). Make clear which confounders were adjusted for and why they were included	Tables 2 and 3 contain unadjusted and progressively adjusted estimates. We also discussed the findings within the text, and provide odds ratios and 95% confidence intervals. We included the confounders based on the literature – we mention this in the paper. As per strobe, we included this information in the methods section; and we omitted repeating this in the results section to reduce redundancy. However, if the editor would like us to repeat this information in the results, we are happy to do so.

For peer review only - http://bmjopen.bmj.com/site/about/guidelines.xhtml

		(*b*) Report category boundaries when continuous variables were categorized	The age cut-offs are provided. In regards to the Townsend index, the methods section states that those below and above the cut-point of zero were compared. In regards to the SOC, those above and below the cut-point of 2 were split.
		(*c*) If relevant, consider translating estimates of relative risk into absolute risk for a meaningful time period	
Other analyses	17	Report other analyses done—eg analyses of subgroups and interactions, and sensitivity analyses	Multiple imputations for missing data were carried out and results are reported. We also reported the results for the test of interaction for GAD and SOC.
**Discussion**			
Key results	18	Summarise key results with reference to study objectives	345-358
Limitations	19	Discuss limitations of the study, taking into account sources of potential bias or imprecision. Discuss both direction and magnitude of any potential bias	383-393, 396-417
Interpretation	20	Give a cautious overall interpretation of results considering objectives, limitations, multiplicity of analyses, results from similar studies, and other relevant evidence	464-481 (We also have a section comparing our study results to those of others: 421-452 as well as a section on potential mechanisms explaining our findings: 454-462)
Generalisability	21	Discuss the generalisability (external validity) of the study results	396-400
**Other information**			
Funding	22	Give the source of funding and the role of the funders for the present study and, if applicable, for the original study on which the present article is based	492-493

*Give information separately for cases and controls in case-control studies and, if applicable, for exposed and unexposed groups in cohort and cross-sectional studies.

Note: An Explanation and Elaboration article discusses each checklist item and gives methodological background and published examples of transparent reporting. The STROBE checklist is best used in conjunction with this article (freely available on the Web sites of PLoS Medicine at http://www.plosmedicine.org/, Annals of Internal Medicine at http://www.annals.org/, and Epidemiology at http://www.epidem.com/). Information on the STROBE Initiative is available at www.strobe-statement.org.

For peer review only

For peer review only - http://bmjopen.bmj.com/site/about/guidelines.xhtml

BMJ Open

Sense of coherence as a coping mechanism for women with anxiety living in deprivation: British population study

Journal:	*BMJ Open*
Manuscript ID	bmjopen-2017-018501.R1
Article Type:	Research
Date Submitted by the Author:	21-Nov-2017
Complete List of Authors:	Remes, Olivia; University of Cambridge, Public Health and Primary Care<br>Wainwright, Nicholas; University of Cambridge, UK, Public Health and Primary Care (retired)<br>Surtees, Paul; University of Cambridge, Department of Public Health and Primary Care<br>LaFortune, Louise; University of Cambridge, Institute of Public Health<br>Khaw, Kay-Tee; University of Cambridge, Department of Public Health and Primary Care<br>Brayne, Carol; University of Cambridge, Institute of Public Health
**Primary Subject Heading**:	Mental health
Secondary Subject Heading:	Epidemiology, Public health
Keywords:	EPIDEMIOLOGY, MENTAL HEALTH, Anxiety disorders < PSYCHIATRY, PUBLIC HEALTH

SCHOLARONE™
Manuscripts

only

For peer review only - http://bmjopen.bmj.com/site/about/guidelines.xhtml

Sense of coherence as a coping mechanism for women with anxiety living in deprivation: British population study

Olivia Remes[1], Nick Wainwright[2], Paul Surtees[1], Louise Lafortune[1], Kay-Tee Khaw[3], Carol Brayne[1]

[1]Department of Public Health and Primary Care, University of Cambridge, Forvie Site, Robinson Way, Cambridge, UK CB2 0SR

[2](Retired) Department of Public Health and Primary Care, University of Cambridge, 2 Worts' Causeway, Cambridge, UK CB1 8RN

[3]Department of Public Health and Primary Care, University of Cambridge, 2 Worts' Causeway, Cambridge, UK CB1 8RN

Figures: 1; Tables: 3; Appendix: 2

Correspondence to: Olivia Remes or260@medschl.cam.ac.uk, telephone: 0777674550

For peer review only

Abstract

OBJECTIVE
Many patients receiving medical treatment for anxiety relapse or do not improve. Research has therefore been turning to coping mechanisms as a way to decrease anxiety rates. Previously, we showed that living in a deprived area significantly increases the risk of generalized anxiety disorder (GAD) in women, but not in men. The objective of this study will be to assess whether sense of coherence (coping mechanism) buffers the influence of area deprivation on women's risk of GAD using data from the European Prospective Investigation of Cancer-Norfolk.

DESIGN
Large, population study.

SETTING
UK population-based cohort.

PARTICIPANTS
30,445 people over the age of 40 were recruited through general practice registers in England. Of these, 20,921 completed a structured health and lifestyle questionnaire used to assess generalised anxiety disorder and sense of coherence. Area deprivation was measured using 1991 Census data, and sense of coherence and anxiety were examined in 1996-2000. 10,277 women had complete data on all covariates.

MAIN OUTCOME MEASURE
Past-year generalised anxiety disorder defined according to the Diagnostic and Statistical Manual of Mental Disorders, fourth edition.

RESULTS
In this study, 2.5% (261/10,277) of women had generalized anxiety disorder. In those with a strong sense of coherence, area deprivation was not significantly associated with anxiety (OR=1.42, 95% CI: 0.86, 2.37). However, among women with a weak sense of coherence, those living in deprived areas had an 88% higher likelihood of having generalized anxiety disorder compared to those living in more affluent areas (OR=1.88, 95% CI: 1.28, 2.77).

CONCLUSION
The absolute number of women living in deprived conditions is large worldwide, and significant numbers are affected by generalized anxiety disorder. Sense of coherence moderates the association between area deprivation and anxiety in women; therefore, interventions targeting coping mechanisms may need to be considered for people with anxiety.

Key words: Anxiety, anxiety disorders, risk factors, gender

For peer review only

Article summary

Strengths and limitations of this study

- We used a large, population-based sample of middle- and older-aged adults and adjusted for a range of important confounders, such as, sociodemographic factors and medical history.

- We used a structured, self-reported questionnaire to assess presence of past-year GAD, and sense of coherence.

- We measured area deprivation and sense of coherence by using common, valid and theoretically-sound indices.

- Those who participated in this study were somewhat less deprived and healthier than individuals living in other parts of England; therefore, our results may not generalize to people living in extremely deprived circumstances.

For peer review only

Introduction

Generalized anxiety disorder[1] is one of the most common anxiety disorders in the general population.[2-4] It is characterized by excessive and pervasive worry about a number of areas of life, and associated symptoms, such as, restlessness, irritability, muscle tension, sleep difficulties, and concentration problems.[1] If left untreated, this disorder can increase the risk for disability, impairment, and suicide.[2-5] Although treatment for anxiety exists in the form of psychotherapy and pharmacotherapy, very few people who need treatment actually receive it.[6] One of the reasons for this is that physicians under- and misdiagnose those affected, and few people experiencing symptoms seek help from the clinician.[7] Low rates of help-seeking is a result of low general awareness about the disorder and treatment options, and people perceiving their anxiety to be an intractable personality trait, rather than a condition that can be treated. These problems are further compounded by the fact that even after patients are treated, many relapse, while some do not experience improvement in symptoms.[7]

While it is not known what causes anxiety, most studies on risk have focused on individual-level determinants of anxiety disorders such as personal income, education and history of psychopathology.[8-11] However, research has shown that the environment can have a profound effect on mental health, over and above individual-level circumstances. The living context, such as, living in a deprived area, can have harmful effects for mental health independently of personal socio-economic status and lifestyle factors.[12,13] Women have been reported to be particularly affected by their context or the environment in which they are living.[14,15] Women living in poor areas seem to be disproportionately affected by mental disorders.[16, 17]. Previously, we showed that women living in deprivation had a

For peer review only

significantly higher risk of generalized anxiety disorder, while this was not observed in men.[16] If women are living in an area with low socio-economic circumstances, they are more likely to be exposed to the stress and strain that arises from deprivation.[14] Exposure to stress can then increase the risk for central nervous system dysfunction and hypothalamic-pituitary-adrenal axis dysregulation, which may lead to the development of GAD.[18,19]

To reduce the risk of mental disorders among women exposed to disadvantage or adversity, coping skills need to be considered. In particular, sense of coherence (SOC), which is a way of viewing life as predictable, manageable, and meaningful, can lower the risk for poor health outcomes.[20,21] Also, SOC is a flexible and adaptive dispositional orientation which enables coping with stressful situations.[21,22]

Two systematic reviews[20,23] showed that SOC is linked to quality of life. A strong SOC is related to good physical and self-perceived health, and is negatively associated with anxiety, depression, and post-traumatic stress disorder (PTSD).[23] In the European-Prospective Investigation of Cancer-Norfolk (EPIC-Norfolk) study of over 18,000 people, a strong SOC contributed to a 20% reduction in all-cause mortality in adults.[24] SOC has also been shown to moderate the influence of disadvantage on mental health outcomes. In a study of people who had faced early childhood deprivation and trauma during the Holocaust, sense of coherence moderated the association between early-life deprivation and posttraumatic stress in old age.[25] A strong SOC can therefore be a major coping resource for preserving health.

For peer review only

Previously[16], we have shown that women living in deprived areas were at increased risk for generalized anxiety disorder. The stress of living in deprivation was harmful for women's mental health, while this association with deprivation was not apparent in men. For this reason, this study will focus on women. The objective of this study will be to determine whether SOC moderates the link between area deprivation and generalized anxiety disorder in women using a large, longitudinal, population cohort.

For peer review only

Methods

Study population

Data were drawn from the population-based European Prospective Investigation of Cancer (EPIC)-Norfolk, described in detail elsewhere[26]. Between 1993 and 1997, 30,445 participants over the age of 40-74 years living in Norwich and the surrounding towns and rural areas were identified through general practice age-sex registers (77,630 people were initially invited to join EPIC-Norfolk). At baseline (1993-97), 30,445 participants consented to join the study and completed a postal Health and Lifestyle (HLQ) questionnaire that captured information on sociodemographics, including age, gender, marital status, highest educational attainment, socio-economic status, and self-reported physician diagnoses of physical diseases. Using participants' postal codes, a measure of area deprivation was derived based on the 1991 Census.[27] Between 1993 and 2000, participants completed self-reported postal questionnaires provided they: 1) were still alive, 2) did not ask to be removed from the study's mailing list, and 3) had a valid mailing address.

All participants recruited through general-practice registers and who completed a baseline health questionnaire were eligible to be included in our study; those who completed a psychosocial questionnaire during follow-up were eligible to be included in our analysis.

Assessment of generalized anxiety disorder (GAD) – outcome

In 1996-2000, 20,921 men and women completed a Health and Life Experiences Questionnaire (HLEQ)[28] used to derive a diagnosis of DSM-IV generalized anxiety disorder (GAD). The primary outcome in this study was past-year GAD. The HLEQ captured the onset

For peer review only

and offset timings of episodes of GAD.[29] Past-year GAD consisted of at least one episode that had offset within 12 months of administration of the HLEQ. DSM-IV GAD was diagnosed if participants reported having uncontrollable, excessive worry for six months or longer on most days than not that resulted in disability or impairment. In addition, at least three of the following symptoms needed to have been present: restlessness, irritability, muscle tension, fatigue, trouble concentrating because of worry, mind going blank, trouble falling asleep, trouble staying asleep, and feeling keyed up or on edge. Of those who completed the HLEQ, 462 met criteria for past-year DSM-IV GAD.

Assessment of potential confounders

Covariates were chosen a priori based on previous literature (their links to anxiety[30-33] and deprivation[34,35]). The baseline Health and Lifestyle Questionnaire (HLQ) was used to ascertain age, gender, education (highest level of education attained: no qualifications, educated to age 16 years, educated to age 18 years, or educated to degree level), marital status (single, married, widowed, separated, divorced), social class (professionals, managerial and technical occupations, skilled workers divided into non-manual and manual, partly skilled workers and unskilled manual workers), and self-reported physician diagnoses of major medical conditions (self-reported stroke, myocardial infarction, and cancer).

The HLEQ was used to determine presence of DSM-IV major depressive disorder. Disability measures based on the SF-36 were also derived for participants using the HLEQ. To determine disability levels, we used the physical component summary score (PCS) of the Medical Outcomes Study 36-Item Short Form (SF-36), a widely-used, validated self-

For peer review only

assessment tool. A score of 100 represents no disability and 0 represents a high level of disability.[36] PCS scores were dichotomized above and below the median.

Assessment of area deprivation – exposure

To examine area deprivation, we used the Townsend Index.[37,38] This index is a composite measure of four variables obtained from the 1991 Census: 1) percentage of economically active residents over age 16 who are unemployed, 2) percentage of households that do not possess a car, 3) percentage of private households that are not owner occupied, and 4) percentage of private households that are overcrowded (have more than 1 person per room). These variables were obtained at the level of the enumeration district. For each variable, Z scores were obtained by dividing the mean by the standard deviation (across enumeration districts in England). The Z-values of the four variables were added together to produce a Townsend index score. Positive values of the index indicate areas that are more deprived, while negative values indicate areas that are less deprived; 0 represents the national mean. The postal codes of participants were record linked to enumeration districts, and participants were considered to live in deprived areas depending on the Townsend index score assigned to their enumeration district.

For peer review only

Ascertainment of sense of coherence

The HLEQ included a three-item SOC questionnaire[39] that assessed each of the SOC constructs. The following questions were used to assess each construct:

Comprehensibility:

Do you usually feel that the things that happen to you in your daily life are hard to understand?

Manageability:

Do you usually see a solution to problems and difficulties that other people find hopeless?

Meaningfulness:

Do you usually feel that your daily life is a source of personal satisfaction?

Participants were given the choice of responding to these questions with yes, usually; yes, sometimes; and no. Comprehensibility was reverse scored, and all items were then summed to provide a total SOC scale ranging from 0 to 6. Higher scores represent weaker SOC.

For peer review only

Statistical analysis

Characteristics of the participants were compared by GAD status. We used correlated data analysis to assess the association between individual- and area-level risk factors of GAD in women and men, separately. A population-average model was constructed, which accounted for the potential correlation introduced by the clustering of individuals within enumeration districts. To estimate the population-average effect of the risk factors of interest on past-year GAD, we used generalized estimating equations. As past-year GAD represents a binary outcome (yes/no) and the intra-cluster correlation is assumed to be equal, GEE with a logit link and an exchangeable correlation structure was used. Adjusted odds ratios (OR) and 95% confidence intervals based on robust standard errors were estimated. Since the number of observations per cluster for those with low and high SOC was too small, standard multivariate logistic regression was conducted and results of this paper are based on the latter.

Individual-level measures consisted of demographic and socio-economic status variables, whereas the area-level measure was the Townsend index. Townsend index scores were used to create a dichotomous variable, with 0 as the cut-point (representing the national average).

SOC was split at the median (of 2) and participants below this cut-point were classified as weak on SOC, while those above this cut-point had a strong SOC. The interaction between area deprivation and SOC in women was assessed. After this, analyses were conducted separately for those with strong and weak levels of SOC. First, unadjusted effect estimates were determined. Next, models were constructed that adjusted for 1) age, social class,

For peer review only

educational attainment; then for 2) age, social class, educational attainment, lifetime history of MDD; and finally for 3) age, social class, educational attainment, lifetime history of MDD, physical diseases, and disability level. Age was assessed as a categorical variable. Models were constructed for participants with complete measurements on all covariates. The brackets show the reference categories that were used for each categorical variable when it was entered in the models - deprivation: no [ref] vs. yes; GAD: no [ref] vs. yes; education: high [ref] vs. low; marital status: married [ref] vs. not married; social class: non-manual [ref] vs. manual; lifetime MDD: no [ref] vs. yes; prevalent physical disease: no [ref] vs. yes; disability level: low [ref] vs. high. These reference categories were based on the literature. Choosing other groupings for the potential confounders would not have changed the results. It was not possible to group the GAD variable otherwise, and area deprivation was analysed in accordance with the literature.

To arrive at the study size, we went through the following steps: of the 30,445 who completed the baseline HLQ, we retained those participants (both men and women) who completed the HLEQ (20,921), and of these, we kept only women with complete data on all covariates (10,277). (Figure 1)

Patient involvement

There were no patients involved in the development of the research question and outcome measures, the design of the study, or the recruitment to and conduct of the study.

For peer review only

Results

77,630 people from general practices in Norfolk were invited to take part in the study, and of these, 30,445 consented.[26] The characteristics of responders versus non-responders are compared in Appendix 1; compared to non-responders, those who took part consisted of slightly more women and slightly younger participants. Of the 30,445 people recruited at baseline, 20,921 completed the HLEQ during follow-up.[26,29] Of those who completed the HLEQ, 10,277 women were retained for analysis in this study, because they had complete data on all covariates. The number of missing observations for each covariate was: 1 for age, 7 for education, 23 for marital status, 303 for social class, 35 for employment, 46 for Townsend index, 883 for disability, 321 for MDD, and 300 for GAD. Participants were assessed between 1993 and 2000 (followed for 7 years) (Figure 1).

In 1996-99, GAD was present in 261 out of 10,277 (2.5%) women. Table 1 shows sociodemographic, medical history, and lifestyle characteristics for women with a weak and strong SOC.

For peer review only

Table 1: Distribution of characteristics for women (n=10,277) with weak and strong SOC who completed the HLEQ questionnaire in the EPIC-Norfolk cohort

	Weak SOC		Strong SOC	
**Characteristic**	**Number with characteristic**	**Percentage and number with past-year GAD**	**Number with characteristic**	**Percentage and number with past-year GAD**
**Socio-demographics**				
**Age (years)**				
<65	1997	6.9 (137)	4768	1.6 (78)
>=65	996	2.7 (27)	2516	0.8 (19)
**Education**[‡]				
Low	1359	4.6 (62)	2668	0.8 (21)
High	1634	6.2 (102)	4616	1.7 (76)
**Marital status**				
Married	2062	5.5 (114)	5651	1.2 (69)
Not married[*]	931	5.4 (50)	1633	1.7 (28)
**Social class**[¥]				
Manual	1262	4.9 (62)	2555	1.1 (27)
Non-manual	1731	5.9 (102)	4729	1.5 (70)
**Employed**				
Yes	1180	5.7 (67)	2875	1.4 (40)
No	1813	5.4 (97)	4409	1.3 (57)
**Townsend index**				
**Deprivation**				
Yes (>0)	534	8.4 (45)	1103	1.7 (19)
No (<=0)	2459	4.8 (119)	6181	1.3 (78)
**Health status**				
**Prevalent physical disease**[+]				
Yes	1684	6.2 (104)	3976	1.8 (70)
No	1309	4.6 (60)	3308	0.8 (27)
**Disability level**				
High[¶]	1718	6.3 (108)	3544	1.8 (64)
Low	1275	4.4 (56)	3740	0.9 (33)
**Lifetime MDD**				
Yes	738	14.0 (103)	1190	5.4 (64)
No	2255	2.7 (61)	6094	0.5 (33)

[+] Prevalent physical disease: respiratory disease (asthma and bronchitis), allergies (allergies and hay fever), stroke, heart attack, cancer, diabetes, thyroid conditions, arthritis

[¥] Manual: skilled manual, semi-skilled, non-skilled; non-manual: professionals, managerial, skilled non-manual

[‡] High education: O-level, A-level, degree; low education: refers to no education

[*] Other: divorced, separated, widowed

[¶] Below the median PCS value of 50.6

For peer review only

Among women with a weak SOC, those who also had GAD were more likely to be younger, have high educational attainment, live in areas of high deprivation, and have had pre-existing health conditions or show prevalent physical disease, high disability, and lifetime MDD. In the group with strong SOC, similar patterns were found as for those with anxiety.

During the 6-year follow-up period, there were a total of 261 GAD cases in women. A weak SOC was found in 2993 women, while a strong SOC was present in 7284 women. When the interaction between area deprivation and SOC was assessed, the p-value was 0.221. Tables 2 and 3 show the unadjusted (Model A) and adjusted odds ratios (Models B-E) associated with GAD in those with a weak and strong SOC, respectively.

For peer review only

Table 2: Odds ratios for women with a weak SOC who completed the HLEQ questionnaire in 1996-00 (women with weak SOC sample size=2993)

	Odds ratios (OR) and 95% CI				
**Characteristic**	**Unadjusted**	**Model A[1]**	**Model B[2]**	**Model C[3]**	**P-value for Model C**
**Socio-demographics**					
**Age**					
<65	2.64 (1.74, 4.02)	3.34 (2.11, 5.30)	2.48 (1.54, 3.97)	2.67 (1.66, 4.29)	<0.0001
>=65	1.00	1.00	1.00	1.00	
**Education[‡]**					
Low	0.72 (0.52, 0.99)	0.76 (0.54, 1.08)	0.82 (0.58, 1.18)	0.82 (0.57, 1.18)	0.288
High	1.00	1.00	1.00	1.00	
**Marital status**					
Married	1.00	1.00	1.00	1.00	
Not married[*]	0.97 (0.69, 1.37)	1.05 (0.73, 1.50)	0.85 (0.59, 1.23)	0.84 (0.58, 1.22)	0.362
**Social class[¥]**					
Manual	0.83 (0.60, 1.14)	0.81 (0.57, 1.14)	0.83 (0.58, 1.17)	0.80 (0.56, 1.14)	0.216
Non-manual	1.00	1.00	1.00	1.00	
**Employed**					
Yes	1.00	1.00	1.00	1.00	
No	0.94 (0.68, 1.29)	1.53 (1.08, 2.16)	1.40 (0.98, 2.01)	1.31 (0.91, 1.88)	0.145
**Townsend index**					
**Deprivation**					
Yes (>0)	1.81 (1.27, 2.59)	2.04 (1.41, 2.96)	1.97 (1.34, 2.88)	1.99 (1.35, 2.92)	0.001
No (<=0)	1.00	1.00	1.00	1.00	
**Health status**					
**Lifetime MDD**					
Yes	5.83 (4.20, 8.11)		5.26 (3.76, 7.35)	5.11 (3.65, 7.16)	<0.0001
No	1.00		1.00	1.00	
**Prevalent physical disease[+]**					
Yes	1.37 (0.99, 1.90)			1.21 (0.85, 1.71)	0.292
No	1.00			1.00	
**Disability level**					
High[¶]	1.46 (1.05, 2.03)			1.51 (1.06, 2.16)	0.023
Low	1.00			1.00	

For peer review only

1. Adjusted for age, SES (education, marital status, social class)
2. Adjusted for age, SES, lifetime MDD
3. Adjusted for age, SES, lifetime MDD, physical disease and disability

† Prevalent physical disease: respiratory disease (asthma, bronchitis), allergies (allergies, hay fever), stroke, heart attack, cancer, diabetes, thyroid conditions, arthritis

¥ Manual: skilled manual, semi-skilled, non-skilled; non-manual: professionals, managerial, skilled non-manual

‡ High education: O-level, A-level, degree; low education: refers to no education

* Other: divorced, separated, widowed

¶ Below the median PCS value of 50.6

For peer review only

Table 3: Odds ratios for women with a strong SOC who completed the HLEQ questionnaire in 1996-00 (women with a strong SOC sample size =7284)

	Odds ratios (OR) and 95% CI				
**Characteristic**	**Unadjusted**	**Model A[1]**	**Model B[2]**	**Model C[3]**	**P-value for Model C**
**Socio-demographics**					
**Age**					
<65	2.19 (1.32, 3.62)	2.61 (1.50, 4.56)	1.93 (1.09, 3.41)	2.17 (1.22, 3.86)	0.009
>=65	1.00	1.00	1.00	1.00	
**Education[‡]**					
Low	0.47 (0.29, 0.77)	0.54 (0.32, 0.90)	0.59 (0.35, 0.99)	0.59 (0.35, 1.00)	0.049
High	1.00	1.00	1.00	1.00	
**Marital status**					
Married	1.00	1.00	1.00	1.00	
Not married	1.41 (0.91, 2.20)	1.56 (0.99, 2.46)	1.24 (0.78, 1.98)	1.21 (0.76, 1.94)	0.429
**Social class[¥]**					
Manual	0.71 (0.46, 1.11)	0.84 (0.52, 1.33)	0.86 (0.53, 1.38)	0.83 (0.51, 1.34)	0.440
Non-manual	1.00	1.00	1.00	1.00	
**Employed**					
Yes	1.00	1.00	1.00	1.00	
No	0.93 (0.62, 1.40)	1.46 (0.94, 2.27)	1.44 (0.92, 2.26)	1.25 (0.79, 1.97)	0.348
**Townsend index**					
**Deprivation**					
Yes (>0)	1.37 (0.83, 2.27)	1.43 (0.86, 2.38)	1.31 (0.78, 2.21)	1.28 (0.76, 2.16)	0.351
No (<=0)	1.00	1.00	1.00	1.00	
**Health status**					
**Life-time MDD**					
Yes	10.44 (6.83, 15.96)		9.35 (6.08, 14.39)	8.62 (5.59, 13.29)	<0.0001
No	1.00		1.00	1.00	
**Prevalent physical disease[+]**					
Yes	2.18 (1.39, 3.40)			1.73 (1.09, 2.74)	0.020
No	1.00			1.00	
**Disability level**					
High[¶]	2.07 (1.35, 3.15)			1.92 (1.23, 3.00)	0.004
Low	1.00			1.00	

For peer review only - http://bmjopen.bmj.com/site/about/guidelines.xhtml

1. Adjusted for age, SES (education, marital status, social class)
2. Adjusted for age, SES, lifetime MDD
3. Adjusted for age, SES, lifetime MDD, physical diseases and disability

[+] Prevalent physical disease: respiratory disease (asthma, bronchitis), allergies (allergies, hay fever), stroke, heart attack, cancer, diabetes, thyroid conditions, arthritis

[¥] Manual: skilled manual, semi-skilled, non-skilled; non-manual: professionals, managerial, skilled non-manual

[‡] High education: O-level, A-level, degree; low education: refers to no education

[*] Other: divorced, separated, widowed

[¶] Below the median PCS value of 50.6

For peer review only

Analyses that adjusted for age, education, marital status, social class, and employment status showed that area deprivation was significantly associated with increased risk for GAD in women with a weak SOC (OR=1.93, 95% CI: 1.34, 2.78) (table 2), but area deprivation was not significantly associated with anxiety in those with strong SOC (OR=1.42, 95% CI: 0.86, 2.37) (table 3). In women with a weak SOC (table 2), further adjustment for lifetime MDD slightly attenuated the effect estimate, though the association between area deprivation and anxiety remained highly significant (OR=1.86, 95%: 1.27, 2.74). When prevalent physical disease and disability level were added to the final model, the effect estimate remained almost unchanged compared to the previous model; among women with poor coping skills, those living in deprived areas had an 88% higher likelihood of having anxiety than women living in less deprived areas (OR=1.88, 95% CI: 1.28, 2.77). For women with a strong SOC (table 3), area deprivation was associated with a small increased risk of having GAD in progressively adjusted models; however, none of the effect estimates reached statistical significance. In the fully-adjusted model, women with a strong SOC and living in deprivation had a 22% higher chance of having GAD compared to women living in less deprived areas, but this did not reach statistical significance (OR=1.28, 95% CI: 0.76, 2.16).

We carried out multiple imputations for missing data (Appendix 2); the effect estimate became even stronger for women with a weak SOC and living in deprivation (OR=2.28, 95% CI: 1.61, 3.23), and the association between deprivation and anxiety become even weaker for women with a strong SOC (OR=1.13, 95% CI: 0.68, 1.90).

For peer review only

Discussion

In this large, population-based study, we found that area deprivation significantly increased the risk for generalized anxiety disorder (GAD) in women, but particularly in those with poor coping skills. Coping skills or sense of coherence (SOC) appeared to moderate the association between area deprivation and anxiety. Women living in deprivation and with poor coping or a weak SOC were at a particularly high risk for having anxiety after controlling for important confounders. Although women with a strong SOC showed a slight increased risk of anxiety if living in disadvantaged circumstances, the association between area deprivation and GAD was statistically non-significant in women who were able to cope well and the effect estimate was much smaller than that of the former group (women with poor coping). A statistically significant association between area deprivation and GAD persisted in women with a weak SOC after adjustment for age, marital status, education level, social class, major depressive disorder, chronic physical diseases, and disability. In contrast, having a strong SOC seemed to be protective for women living in deprived areas. Having a strong SOC rendered the association between area deprivation and anxiety statistically non-significant.

Although the interaction between area deprivation and SOC was not statistically significant, the effect estimates do suggest that there are differences between women with low and high SOC. Our study sheds light on the importance of SOC when it comes to mitigating the risks of anxiety. Future research should replicate our study with a larger number of anxiety cases, perhaps by measuring 'total' or 'any' anxiety rather than individual disorders, such as GAD.

For peer review only

Deprived areas are often associated with low social integration and poor social control. Emile Durkheim showed that low social integration can lead to a sense of meaninglessness among individuals, and this can give rise to poor mental health and suicide.[40] SOC is a way of viewing life as meaningful and comprehensible, and our study shows that SOC can moderate the association between area deprivation and GAD in women.

For peer review only

Strengths and limitations of this study, and future research

This is the largest, population-based study of the association between area deprivation and GAD in women, and to determine whether coping resources or SOC mediates the association between area deprivation and anxiety. We had access to a large sample of over 10,000 women living in the community. We used a clinically relevant measure of anxiety, and GAD was defined according to the Diagnostic and Statistical Manual of Mental Disorders, fourth version (DSM-IV). Although GAD affects a substantial number of people, even more experience subthreshold cases of anxiety disorders. Subthreshold cases have also been associated with impairment and disability; therefore, future research should assess associations with subclinical anxiety.

We used detailed health and lifestyle questionnaires to extract information on demographics, social class, and major chronic physical diseases, and controlled for these factors in our analyses. We used a validated and reliable measure of disability, which we adjusted for in our models. We had a large list of self-reported physician diagnoses that we used to establish medical histories for participants, though three issues might arise with this approach. First, the residual effect of diseases not captured by our study but that are associated with area deprivation and anxiety, may be present. Second, medical diagnoses were not verified by clinicians, leading to possible misclassification. Third, past illness may have been under-reported, leading to misclassification bias and attenuation of effect estimates. We may have overadjusted our models with the inclusion of disability, because this might be part of the expression of psychiatric illness. This may have reduced effect estimates. Our objective was to assess the links between deprivation, SOC, and anxiety in women. Although it was out of scope for the present study, we were unable to examine the

For peer review only

same objectives in men: there were very few men with a strong SOC living in deprivation and with GAD. Therefore, analyses in this sub-group would not have been robust. Future studies should undertake this assessment. It should also be mentioned that the internal consistency of the three-items SOC scale, as measured by Chronbach's alpha, was 0.35.[22] While the internal consistency of the shorter 3-item measure was low in this sample, this is likely to be partially due to the small number of scale items. Also, the original developers of the scale reported satisfactory short-term test-retest reliability and validity for the 3-item measure.[22,41]

At baseline, people who consented to take part in EPIC-Norfolk agreed to fill out detailed health and lifestyle questionnaires over the duration of the study period; therefore, healthy volunteer effect may have biased our findings. Participants in EPIC-Norfolk tend to be somewhat healthier and more affluent than the general population, therefore, results from this study cannot be generalized to extremely deprived areas. If the most deprived areas would have been included, we would expect the association between area deprivation and anxiety to be even stronger in women with a weak SOC. Also, when comparing the demographic characteristics of responders versus non-responders (Appendix 1), we found that participants were slightly younger and slightly more women than men consented.

Also, it may be that participants with poorer mental health may have moved to more deprived neighbourhoods; however, reverse causality seems unlikely as an explanation for our findings. In addition, deprivation was measured before anxiety in this study; however, SOC was examined at the same time point as GAD, rendering this study cross-sectional.

For peer review only

Non-participation in our study may have contributed to non-differential misclassification and attenuation of effect estimates. Although our study is observational in nature and cannot confirm causality between area deprivation and generalized anxiety disorder in women with a strong and weak SOC, a rigorous analysis based on observational data is a reasonable way of examining this relationship. When we conducted multiple imputations, the effect estimate for women with a weak SOC became even greater, and among women with a strong SOC, it attenuated towards the null. Our study provides a valuable step forward and is the first to shed light on the importance of coping in people with GAD living in disadvantaged circumstances.

Comparison with other studies

This is the largest, population-based study to consider the association between area deprivation and GAD in women, and to determine whether SOC mediates this association. Most of the literature on coping and sense of coherence is limited. Most studies have small sample sizes, and measure people's coping abilities in relation to feelings of stress, history of stressful life events, or exposure to stressful circumstances, such as, wars. There is a paucity of research examining the living context, such as, area deprivation, and no studies have assessed whether the link between area-level circumstances and anxiety disorders can be mediated by coping mechanisms. The literature on coping uses highly select samples; therefore, results cannot be generalized to the larger population. Also, incomplete adjustment of covariates makes it difficult to determine whether findings from these studies are not better explained by the residual effect of other factors that have not been accounted for, such as, lifestyle and personal socio-economic circumstances. Across studies, there is large heterogeneity in the definitions used to define coping, with many focusing on

factors, such as, hardiness, optimism, and negative emotions, rather than SOC. In sum, it is difficult to understand the links between the living context, coping abilities, and mental health from the literature; however, the studies that have been conducted are a good starting point.

A UK study of over 3000 people [42] showed that SOC was linked to self-rated health; however, the moderating effect of coping was not assessed. Research on people living in Negev communities in Israel showed that those exposed to trauma and severe stress-provoking situations, but who had a strong SOC, were least likely to develop stress.[43] In a study of French adults [44], SOC buffered the effect of adversity on psychological well-being. In another study of Holocaust survivors [25], SOC moderated the association between early childhood deprivation and posttraumatic stress in old age. Both of these latter studies, however, were small, failed to adjust for important confounders, such as sociodemographic factors and disability, and did not examine individual psychiatric disorders diagnosed according to valid and reliable criteria, such as, the DSM. In the study on child Holocaust survivors [25], exposure to trauma was measured in early life, while posttraumatic stress in old age. Since participants were required to report traumas experienced in childhood, this might have led to recall bias. Our study expands on previous research and is the first to investigate the moderating effect of coping skills (sense of coherence [SOC]) on the risk of developing generalized anxiety disorder (GAD) in women living in deprived circumstances.

Mechanism of effect

Living in a deprived area can increase anxiety in women because of biological and social factors.[16] The stress of living in deprivation can increase the risk for inflammation and

For peer review only

HPA-axis dysregulation, which can lead to GAD.[18,19,45] This, combined with the multiple roles that women are increasingly taking on (income earner, child-bearer, and carer of elderly relatives)[45], means that coping is particularly relevant for women living in disadvantaged circumstances. A strong SOC is linked to high quality of life, and good physical and mental health.[20,23] Our study shows that SOC can buffer the effect of area deprivation on risk of anxiety.

Implications

The absolute number of people living in deprived conditions is large worldwide, and significant numbers will have been affected by generalized anxiety disorder (GAD).[46] For the first time, we show that sense of coherence (SOC) moderates the association between area deprivation and anxiety in women. Future research should replicate our analysis using larger samples and determine the specific components of SOC that attenuate the effect of deprivation on mental health. Interventions can then be developed to target components of SOC to increase people's coping resources. Treatment for generalized anxiety disorder exists, with psychotherapy and pharmacotherapy being commonly prescribed. However, success rates are fairly low, patients relapse, and some fail to experience any symptom improvement. Costs to the health care system related to anxiety are substantial. Therefore, targeting people's coping resources could represent another option for people with anxiety, including those who do not experience symptom improvement following commonly-prescribed therapies. Targeting SOC could also represent a better option for people who have faced extreme circumstances and adversity, and who may have difficultly dealing with the traumas directly as during psychotherapy. Interventions should take these findings into

For peer review only

account, and mental health policy should also consider improving living environments to decrease the burden of anxiety in women.

Acknowledgements: OR received a PhD studentship from the National Institute for Health Research.

Competing interest: All authors have completed the ICMJE uniform disclosure form at www.icmje.org/coi_disclosure.pdf and declare: no support from any organisation for the submitted work; no financial relationships with any organizations that might have an interest in the submitted work in the previous three years; no other relationships or activities that could appear to have influenced the submitted work.

Funding: This work was supported by the Medical Research Council UK (grant number SP2024-0201 and SP2024-0204) and Cancer Research UK (grant number G9502233).

Author contributions: OR (corresponding author) had the idea for and conducted the analysis, and wrote the article. CB critically reviewed drafts of the manuscript, KK edited versions of the paper; PS and NW provided feedback into the analysis. OR, CB, KK, LL, PS, and NW contributed to the interpretation of data for the work, agreed to be accountable for all aspects of the work, gave final approval of the version to be published, and made substantial contributions to the analysis and interpretation of data. OR, CB, KK, LL, PS, and NW have seen and approved the final version. OR, CB, KK, LL, PS, and NW had full access to all the data in the study and take responsibility for the integrity of the data and the accuracy of the data analysis. OR acts as guarantor of the study.

For peer review only

Transparency declaration: OR affirms that the manuscript is an honest, accurate, and transparent account of the study being reported; that no important aspects of the study have been omitted; and that any discrepancies from the study as planned have been explained.

Role of study sponsors and statement of independence: The funding sources had no role in the design and conduct of the study; collection, management, analysis, and interpretation of the data; and preparation, review, or approval of the manuscript.

Ethical approval: The study has ethics committee approval from Norfolk Ethics Committee (Rec Ref: 98CN01) and all participants gave informed consent.

Data sharing: No additional data available. Original dataset requests should be sent to the corresponding author. Please contact O Remes at or260@medschl.cam.ac.uk for questions about the statistical code.

Copyright: "The Corresponding Author has the right to grant on behalf of all authors and does grant on behalf of all authors, a worldwide licence to the Publishers and its licensees in perpetuity, in all forms, formats and media (whether known now or created in the future), to i) publish, reproduce, distribute, display and store the Contribution, ii) translate the

For peer review only

Contribution into other languages, create adaptations, reprints, include within collections and create summaries, extracts and/or, abstracts of the Contribution, iii) create any other derivative work(s) based on the Contribution, iv) to exploit all subsidiary rights in the Contribution, v) the inclusion of electronic links from the Contribution to third party material where-ever it may be located; and, vi) licence any third party to do any or all of the above."

For peer review only

References

1. American Psychiatric Association. (1994). *Diagnostic and Statistical Manual of Mental Disorders* (4th edn). Washington, DC: APA.
2. Kessler R, Keller M, Wittchen H. The epidemiology of generalized anxiety disorder. *Psychiatr Clin North Am* 2001;24:19–39, doi: http://dx.doi.org/10.1016/S0193-953X(05)70204-5 [published Online First 29 June 2005].
3. Stein MB, Sareen J. Generalized anxiety disorder. *New Engl J Med* 2015;373:2059–2068, doi: 10.1056/NEJMcp1502514 [published Online First 19 November 2015].
4. Hoge E, Ivkovic A, Fricchione G. Generalized anxiety disorder: diagnosis and treatment. *BMJ* 2012;345,e7500, doi: http://dx.doi.org/10.1136/bmj.e7500 [published Online First 27 November 2012].
5. Nepon J, Belik S, Bolton J, Sareen J. The relationship between anxiety disorders and suicide attempts: findings from the National Epidemiologic Survey on Alcohol and Related Conditions. *Depress Anx* 2010;27:791–798, doi: 10.1002/da.20674 [published Online First 9 March 2010].
6. Revicki DA, Travers K, Wyrwich KW. Humanistic and economic burden of generalized anxiety disorder in North America and Europe. *J Affect Disord* 2012;140:103–12.
7. Simpson HB, Neria Y, Lewis-Fernandez R, Schneier F. Anxiety disorders: theory, research and clinical perspectives. Cambridge University Press, 2010.
8. Grant BF, Hasin DS, Stinson FS, et al. Prevalence, correlates, co-morbidity, and comparative disability of DSM-IV generalized anxiety disorder in the USA: results from the National Epidemiologic Survey on Alcohol and Related Conditions. *Psychol Med* 2005;35:1747, doi: 10.1017/S0033291705006069 [published Online First 5 October 2005].

9. The NHS Information Centre for health and social care, Leeds. Adult psychiatric morbidity in England, 2007 Results of a household survey. http://www.esds.ac.uk/doc/6379/mrdoc/pdf/6379research_report.pdf (accessed 29 July 2014).

10. Fryers T, Melzer D, Jenkins R. Social inequalities and the common mental disorders: a systematic review of the evidence. *Soc Psychiatry Psychiatr Epidemiol* 2003;38:229–237, doi: 10.1007/s00127-003-0627-2.

11. Zhang X, Norton J, Carriere I, Ritchie K, Chaudieu I, Ancelin ML. Risk factors for late-onset generalized anxiety disorder: results from a 12-year prospective cohort (the ESPRIT study). *Transl Psychiatry* 2015;5:e536, doi: 10.1038/tp.2015.31 [published Online First 31 March 2015].

12. Subramanian S, Kawachi I. Income inequality and health: what have we learned so far?. *Epidemiol Rev* 2004;26:78–91, doi: 10.1093/epirev/mxh003.

13. Pickett KE, Wilkinson RG. Income inequality and health: a causal review. *Soc Sci Med* 2014;128:316–326, doi: 10.1016/j.socscimed.2014.12.031 [published Online First 30 December 2014].

14. Pattyn E, Van Praag L, Verhaeghe M, Levecque K, Bracke P. The association between residential area characteristics and mental health outcomes among men and women in Belgium. *Arch Public Health* 2011;69:3, doi: 10.1186/0778-7367-69-3 [published Online First 24 October 2011].

15. Stafford M, Cummins S, Macintyre S, Ellaway A, Marmot M. Gender differences in the associations between health and neighbourhood environment. *Soc Sci Med* 2005;60:1681–1692, doi: 10.1016/j.socscimed.2004.08.028 [published Online First 5 November 2004].

For peer review only

16. Remes O, Wainwright N, Surtees P, Lafortune L, Khaw K, Brayne C. Sex differences in the association between area deprivation and generalized anxiety disorder: British population study. *BMJ Open* 2017 (in press).

17. Morrissey K. Gender differences in the association between common mental disorders and regional deprivation in Ireland. *Prof Geogr* 2015;68:129-137 doi: 10.1080/00330124.2015.1054020 [published Online First 24 July 2015].

18. Hoehn-Saric R, McLeod D, Funderburk F, Kowalski P. Somatic symptoms and physiologic responses in generalized anxiety disorder and panic disorder. *Arch Gen Psychiatry* 2004;61:913, doi: 10.1001/archpsyc.61.9.913.

19. Chaudieu I, Beluche I, Norton J, Boulenger JP, Ritchie K, Angelin ML. Abnormal reactions to environmental stress in elderly persons with anxiety disorders: evidence from a population study of diurnal cortisol changes. *J Affect Disord* 2008;106:307–313, doi: 10.1016/j.jad.2007.07.025 [published Online First 28 August 2007].

20. Eriksson M, Lindstrom B. Antonovsky's sense of coherence scale and its relation with quality of life: a systematic review. *J Epidemiol Community Health* 2007;61:938-944, doi: 10.1136/jech.2006.056028.

21. Antonovsky A. Unravelling the mystery of health. How people manage stress and stay well. San Francisco, CA: Lossey-Bass 1987.

22. Surtees PG, Wainwright NW, Khaw KT. Resilience, misfortune, and mortality: evidence that sense of coherence is a marker of social stress adaptive capacity. *J Psychosom Res* 2006;61:221-227, doi: 10.1016/j.jpsychores.2006.02.014.

23. Eriksson M, Lindstrom B. Antonovsky's sense of coherence scale and the relation with health: a systematic review. *J Epidemiol Community Health* 2006;60:376-381, doi: 10.1136/jech.2005.041616.

For peer review only

24. Wainwright NWJ, Surtees PG, Welch AA, Luben RN, Khaw K-T, Bingham SA. Sense of coherence, lifestyle choices and mortality. J Epidemiol Community Health 2008;62:829-831 doi: http://dx.doi.org/10.1136/jech.2007.066464.

25. van der Hal-van Raalte EAM, van IJzendoorn MH, Bakermans-Kranenburg MJ. Sense of coherence moderates late effects of early childhood holocaust exposure. J Clin Psychol 2008;64:1352-67. doi: 10.1002/jclp.20528. [published Online First 24 October 2008].

26. Hayat SA, Luben R, Keevil VL, et al. Cohort profile: a prospective cohort study of objective physical and cognitive capability and visual health in an ageing population of men and women in Norfolk (EPIC-Norfolk 3). *Int J Epidemiol* 2013;1-10 [published Online First 14 June 2013].

27. Office for National Statistics, UK. Census data 1801 1991. https://www.ons.gov.uk/census/2011census/2011censusdata/censusdata18011991 (accessed 20 Apr 2015).

28. Ishihara-Paul L, Wainwright NW, Khaw KT, et al. Prospective association between emotional health and clinical evidence of Parkinson's disease. *Eur J Neurol* 2008;15:1148-54, doi: 10.1111/j.1468-1331.2008.02299.

29. Surtees P, Wainwright N, Khaw K, Day N. Functional health status, chronic medical conditions and disorders of mood. *Br J Psychiatry* 2003;183:299–303, doi: 10.1192/bjp.183.4.299 [published Online First 30 September 2003].

30. Remes O, Brayne C, Van der Linde R, Lafortune L. A systematic review of reviews on the prevalence of anxiety disorders in adult populations. *Brain Behav* 2016;6:e00497 doi: 10.1002/brb3.497 [published Online First 5 June 2016].

For peer review only

31. Weisberg RB. Overview of generalized anxiety disorder: epidemiology, presentation, and course. *J Clin Psychiatry* 2009;70:4-9. doi: 10.4088/JCP.s.7002.01.

32. Wittchen HU. Generalized anxiety disorder: prevalence, burden, and cost to society. *Depress Anxiety* 2002;16:162-71 doi: 10.1002/da.10065 [published Online First 18 December 2002].

33. Culpepper L. Generalized anxiety disorder and medical illness. J Clin Psychiatry 2009;70:20-4.

34. Lakshman R, McConville A, How S, Flowers J, Wareham N, Cosford P. Association between area level socio-economic deprivation and a cluster of behavioural risk factors: Cross sectional, population based study. *J Public Health (Oxf)* 2011;33:234-245. doi: 10.1093/pubmed/fdq072 [published Online First 29 September 2010].

35. Vetter S, Endrass J, Schweizer I, Teng H-M, Rossler W, Gallo WT. The effects of economic deprivation on psychological well-being among the working population of Switzerland. *BMC Public Health* 2006;6:223. doi: 10.1186/1471-2458-6-223 [published Online First 4 September 2006].

36. Ware JE, Snow K, Kosinski M, Gandek B. New England Medical Center Hospital. Health Institute. SF-36 Health Survey: Manual and Interpretation Guide. The Health Institute; Boston, MA: New England Medical Center 1993.

37. National Centre for Research Methods. Townsend deprivation index. http://www.restore.ac.uk/geo-refer/36229dtuks00y19810000.php (accessed 15 April 2014).

38. Shohaimi S, Luben R, Wareham N, et al. Residential area deprivation predicts smoking habit independently of individual educational level and occupational social class. A cross sectional study in the Norfolk cohort of the European Investigation into

For peer review only

Cancer (EPIC-Norfolk). *J Epidemiol Community Health* 2003;57:270–276, doi: 10.1136/jech.57.4.270.

39. Surtees PG, Wainwright NWJ, Luben R, Khaw K-T, Day NE. Mastery, sense of coherence, and mortality: evidence of independent associations from the EPIC-Norfolk prospective cohort study. *Health Psychol* 2006;25:102-10, doi: 10.1037/0278-6133.25.1.102.

40. Durkheim E. Suicide – a study in sociology. Simon and Schuster 2010.

41. Lundberg O, Peck MN. A simplified way of measuring sense of coherence: experiences from a population survey in Sweden. *Eur J Pub Health*; 1995;5:56-59, doi: https://doi.org/10.1093/eurpub/5.1.56.

42. Walsh D, McCartney G, McCullough S, Buchanan D, Jones R. Comparing Antonovsky's sense of coherence scale across three UK post-industrial cities. BMJ Open 2014;4:e005792, doi: http://dx.doi.org/10.1136/bmjopen-2014-005792.

43. Braun-Lewensohn O, Sagy S. Community resilience and sense of coherence as protective factors in explaining stress reactions: comparing cities and rural communities during missiles attacks. *Community Mental Health J* 2014;50:229-234, doi: 10.1007/s10597-013-9623-5 [published Online First 9 June 2013].

44. Gana K. Is sense of coherence a mediator between adversity and psychological wellbeing in adults? *Stress and Health* 2001;17:77-83, doi: 10.1002/smi.882 [published Online First 26 February 2001].

45. Remes O, L Lafortune, K-T Khaw, C Brayne. Sex and gender in psychiatry. Lancet Psychiatry 2017;4:e1, doi: http://dx.doi.org/10.1016/S2215-0366(16)30445-X.

46. Whiteford HA, Degenhardt L, Rehm J, et al. Global burden of disease attributable to mental and substance use disorders: findings from the Global Burden of Disease

For peer review only

Study 2010. *Lancet* 2013;9904:1575-1586 doi: http://dx.doi.org/10.1016/S0140-6736(13)61611-6 [published Online First 29 August 2013].

For peer review only

Figure 1 - Flowchart of EPIC-Norfolk cohort

This is a flowchart showing the number of participants at each study stage: the number approached to participate in the EPIC-Norfolk study, the number enrolled at baseline, and with complete data on all covariates. The EPIC-Norfolk study consists of middle-aged and older British people.

For peer review only

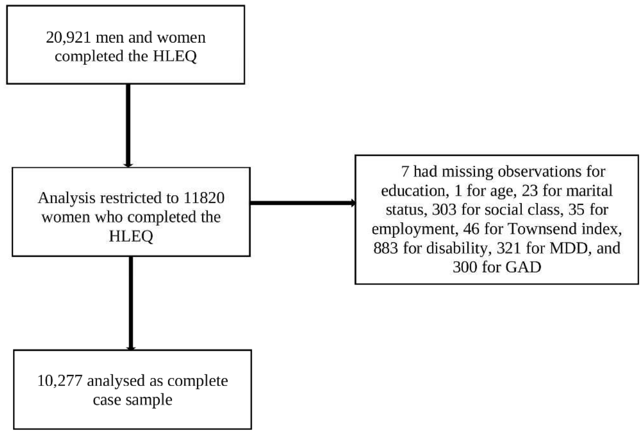

Flow chart of European Prospective Investigation of Cancer (EPIC)-Norfolk cohort. This is a flowchart showing the number of participants enrolled at baseline, and with complete data on all covariates. The EPIC-Norfolk study consists of middle-aged and older British people.

Appendix 1: Characteristics of participants who consented (n=30,445) and refused (n=43,452) to take part in the EPIC-Norfolk cohort study

	Percentage (number)	
**Characteristic**	**Consented**	**Did not consent**
**Age**		
<50	27.5 (8366)	33.7 (14647)
50-60	30.3 (9230)	29.5 (12819)
60-70	32.5 (9879)	27.4 (11898)
>=70	9.8 (2970)	9.4 (4088)
**Gender**		
Female	55.0 (16744)	49.0 (21296)
Male	45.0 (13701)	51.0 (22156)

For peer review only

Appendix 2 – Supplementary Material

We imputed missing data separately for women with a weak and strong sense of coherence (SOC). Based on the literature, we identified 12 potential auxiliary variables; however, we retained eight variables that were correlated with the variables in our model and were good predictors of the missing status (based on statistical tests). Our imputation model included all variables in the analysis model and the auxiliary variables.

To retain as much information as possible, we conducted the imputations on non-transformed data-the original variables in our dataset. We imputed data using the fully conditional specification, and specified a linear regression model for continuous data that were normally distributed; predictive mean matching for continuous data that were not normally distributed; and logistic regression for categorical variables. Variable estimates were subsequently averaged from 100 imputed datasets using Rubin's rules (we transformed the data before running the analytic model of interest within each of the imputed datasets).[1]

We checked whether the imputations were acceptable by comparing 1) the means, standard deviations, and plots of recorded and imputed values for continuous variables, and 2) the frequencies and percentages of recorded and imputed values for each level of categorical variables.

Analyses were done using SAS 9.3 and p-values less than 0·05 were considered statistically significant.

Auxiliary variables used in the imputation model

Variable	Questionnaire	Description of variable
**Psychological factors**		
Paternal affection	Health and Life Experiences Questionnaire (HLEQ)	Self-reported paternal affection using the Rossi scale[2]. The scale assessed items, such as, family composition, parental divorce and death, quality of relationship with the father and amount of affection received.
Mastery	Health and Life Experiences Questionnaire (HLEQ)	Self-reported using the Pearlin and Schooler Mastery Scale. Mastery is having a sense of control over one's life or the belief that one has control over future important life circumstances. It represents a coping resource that people use to manage or attenuate the impact of stressors, and this in turn, has an influence on health and health behaviours.[3,4]
Neuroticism	Health and Life Experiences Questionnaire (HLEQ)	Self-reported using the Eysenck Personality Inventory. A tendency towards experiencing negative, distressing emotions.[5]

For peer review only

**Sociodemographic factors**		
School age	Health and Lifestyle (HLQ) Questionnaire	Self-reported age when participant left school.
**Physical health**		
Systolic blood pressure	Baseline health check	Systolic blood pressure measured using an Accutorr noninvasive oscillometric blood pressure monitor; mean of two measurements in mmHg
Diastolic blood pressure	Baseline health check	Diastolic blood pressure measured using an Accutorr noninvasive oscillometric blood pressure monitor; mean of two measurements in mmHg
History of high cholesterol	Health and Lifestyle Questionnaire (HLQ)	Self-reported history of high cholesterol
History of psychiatric illness	Health and Lifestyle Questionnaire (HLQ)	Self-reported history of other psychiatric illness

The questionnaires used for these variables have been previously described in the methods.

The following auxiliary variables were not included in the imputation model, because they were not correlated with the variables in our model and were not good predictors of the missing status (following tests using Pearson's/Spearman's correlation coefficient and t-tests/chi-square tests): composite measure of maternal affection using the Rossi scale[2], and self-reported history of: migraine, benign tumours, psychiatric illness, and back pain.

References

1. Berglund P, Heeringa S. Multiple imputation of missing data using SAS. Cary, NC: *SAS Institute Inc*, 2014.
2. Rossi AS. Caring and doing for others: social responsibility in the domains of family, work, and community. Chicago: *University of Chicago Press*, 2001.
3. Pearlin LI, Menaghan EG, Lieberman MA, Mullan JT. The stress process. *J Health Soc Behav* 1981;22:337–56.
4. Pearlin LI, Schooler C. The structure of coping. *J Health Soc Behav* 1978;19:2–21.
5. Shipley BA, Weiss A, Der G, Taylor MD, Deary IJ. Neuroticism, extraversion, and mortality in the UK Health and Lifestyle Survey: a 21-year prospective cohort study. *Psychosom Med* 2007;69:923–31 doi: 10.1097/PSY.0b013e31815abf83 [published Online First 8 November 2007].

For peer review only

Please see the article line numbers (column on the right) and the explanations provided.

STROBE Statement—checklist of items that should be included in reports of observational studies

	Item No	Recommendation	Line numbers within the article
**Title and abstract**	1	(*a*) Indicate the study's design with a commonly used term in the title or the abstract	7, 39, 42
		(*b*) Provide in the abstract an informative and balanced summary of what was done and what was found	29-64
**Introduction**			
Background/rationale	2	Explain the scientific background and rationale for the investigation being reported	87-136
Objectives	3	State specific objectives, including any prespecified hypotheses	136-138
**Methods**			
Study design	4	Present key elements of study design early in the paper	142
Setting	5	Describe the setting, locations, and relevant dates, including periods of recruitment, exposure, follow-up, and data collection	142-157, 160-161, 190-191, especially 155-157
Participants	6	(*a*) *Cohort study*—Give the eligibility criteria, and the sources and methods of selection of participants. Describe methods of follow-up<br>*Case-control study*—Give the eligibility criteria, and the sources and methods of case ascertainment and control selection. Give the rationale for the choice of cases and controls<br>*Cross-sectional study*—Give the eligibility criteria, and the sources and methods of selection of participants	142-147, 151-153
		(*b*) *Cohort study*—For matched studies, give matching criteria and number of exposed and unexposed<br>*Case-control study*—For matched studies, give matching criteria and the number of controls per case	
Variables	7	Clearly define all outcomes, exposures, predictors, potential confounders, and effect modifiers. Give diagnostic criteria, if applicable	159-170, 189-200, 172-187, 204-219
Data sources/ measurement	8*	For each variable of interest, give sources of data and details of methods of assessment (measurement). Describe comparability of assessment methods if there is	There were three variables of interest in this study: area deprivation, sense of coherence, and generalized

For peer review only - http://bmjopen.bmj.com/site/about/guidelines.xhtml

		more than one group	anxiety disorder. The others are potential confounders – in the methods I list them all, indicate how they were assessed and mention that they were collected through the baseline, self-reported postal HLQ questionnaire as well as the HLEQ.
Bias	9	Describe any efforts to address potential sources of bias	246, 347
Study size	10	Explain how the study size was arrived at	256-259
Quantitative variables	11	Explain how quantitative variables were handled in the analyses. If applicable, describe which groupings were chosen and why	In the methods, I mention how the variables were derived based on the raw data provided by participants in the questionnaires.<br><br>Dependent variable: 159-170<br>Area-level measure: 189-200<br>Individual-level measures: 172-187<br>Effect modifier: 204-219<br><br>In the text, I mention that the categorization was done in accordance with the literature.
Statistical methods	12	(*a*) Describe all statistical methods, including those used to control for confounding	221-259
		(*b*) Describe any methods used to examine subgroups and interactions	I wanted to determine whether sense of coherence (SOC) was an effect modifier in the association between area deprivation and anxiety. I therefore compared the association between deprivation and anxiety in women with both high and low SOC. Before doing this, I examined the interaction between anxiety and SOC. This is described in the methods.
		(*c*) Explain how missing data were addressed	We indicated that this was a complete-case analysis.
		(*d*) *Cohort study*—If applicable, explain how loss to follow-up was addressed<br>*Case-control study*—If applicable, explain how matching of cases and controls was addressed<br>*Cross-sectional study*—If applicable, describe analytical methods taking account of	Loss to follow-up was not a problem in this study. We were able to track down all participants using various means, unless they expressed that they wished to be removed from the mailing list. We elaborate on

For peer review only - http://bmjopen.bmj.com/site/about/guidelines.xhtml

sampling strategy	this in the manuscript.
(*e*) Describe any sensitivity analyses	Logistic regression replaced GEE, because of statistical considerations. This was discussed in the methods section.

For peer review only

For peer review only - http://bmjopen.bmj.com/site/about/guidelines.xhtml

**Results**			
Participants	13*	(a) Report numbers of individuals at each stage of study—eg numbers potentially eligible, examined for eligibility, confirmed eligible, included in the study, completing follow-up, and analysed	256-259, 270-271
		(b) Give reasons for non-participation at each stage	We do not have the reasons for non-participation, because these data were not collected when the study was initiated in 1993. There is some information comparing responders versus non-responders – Appendix I in our paper.
		(c) Consider use of a flow diagram	Flow diagram included in submission.
Descriptive data	14*	(a) Give characteristics of study participants (eg demographic, clinical, social) and information on exposures and potential confounders	278-280, 291-297; we provided characteristics for those with vs. without GAD, because we felt it was important to show the characteristics of those exposed vs. non-exposed (see also Table 1)
		(b) Indicate number of participants with missing data for each variable of interest	273-275
		(c) *Cohort study*—Summarise follow-up time (eg, average and total amount)	276, 296
Outcome data	15*	*Cohort study*—Report numbers of outcome events or summary measures over time	278, 296-297
		Case-control study—Report numbers in each exposure category, or summary measures of exposure	
		Cross-sectional study—Report numbers of outcome events or summary measures	
Main results	16	(*a*) Give unadjusted estimates and, if applicable, confounder-adjusted estimates and their precision (eg, 95% confidence interval). Make clear which confounders were adjusted for and why they were included	Tables 2 and 3 contain unadjusted and progressively adjusted estimates. We also discussed the findings within the text, and provide odds ratios and 95% confidence intervals. We included the confounders based on the literature – we mention this in the paper. As per strobe, we included this information in the methods section; and we omitted repeating this in the results section to reduce redundancy. However, if the editor would like us to repeat this information in the results, we are happy to do so.

For peer review only - http://bmjopen.bmj.com/site/about/guidelines.xhtml

		(*b*) Report category boundaries when continuous variables were categorized	The age cut-offs are provided. In regards to the Townsend index, the methods section states that those below and above the cut-point of zero were compared. In regards to the SOC, those above and below the cut-point of 2 were split.
		(*c*) If relevant, consider translating estimates of relative risk into absolute risk for a meaningful time period	
Other analyses	17	Report other analyses done—eg analyses of subgroups and interactions, and sensitivity analyses	Multiple imputations for missing data were carried out and results are reported. We also reported the results for the test of interaction for GAD and SOC.
**Discussion**			
Key results	18	Summarise key results with reference to study objectives	353-371
Limitations	19	Discuss limitations of the study, taking into account sources of potential bias or imprecision. Discuss both direction and magnitude of any potential bias	396-430
Interpretation	20	Give a cautious overall interpretation of results considering objectives, limitations, multiplicity of analyses, results from similar studies, and other relevant evidence	482-499 (We also have a section comparing our study results to those of others: 438-470 as well as a section on potential mechanisms explaining our findings: 480-488)
Generalisability	21	Discuss the generalisability (external validity) of the study results	415-417
**Other information**			
Funding	22	Give the source of funding and the role of the funders for the present study and, if applicable, for the original study on which the present article is based	509-510

*Give information separately for cases and controls in case-control studies and, if applicable, for exposed and unexposed groups in cohort and cross-sectional studies.

Note: An Explanation and Elaboration article discusses each checklist item and gives methodological background and published examples of transparent reporting. The STROBE checklist is best used in conjunction with this article (freely available on the Web sites of PLoS Medicine at http://www.plosmedicine.org/, Annals of Internal Medicine at http://www.annals.org/, and Epidemiology at http://www.epidem.com/). Information on the STROBE Initiative is available at www.strobe-statement.org.

For peer review only

For peer review only - http://bmjopen.bmj.com/site/about/guidelines.xhtml

BMJ Open

Sense of coherence as a coping mechanism for women with anxiety living in deprivation: British population study

Journal:	*BMJ Open*
Manuscript ID	bmjopen-2017-018501.R2
Article Type:	Research
Date Submitted by the Author:	13-Feb-2018
Complete List of Authors:	Remes, Olivia; University of Cambridge, Public Health and Primary Care<br>Wainwright, Nicholas; University of Cambridge, UK, Public Health and Primary Care (retired)<br>Surtees, Paul; University of Cambridge, Department of Public Health and Primary Care<br>LaFortune, Louise; University of Cambridge, Institute of Public Health<br>Khaw, Kay-Tee; University of Cambridge, Department of Public Health and Primary Care<br>Brayne, Carol; University of Cambridge, Institute of Public Health
**Primary Subject Heading**:	Mental health
Secondary Subject Heading:	Epidemiology, Public health
Keywords:	EPIDEMIOLOGY, MENTAL HEALTH, Anxiety disorders < PSYCHIATRY, PUBLIC HEALTH

SCHOLARONE™
Manuscripts

For peer review only - http://bmjopen.bmj.com/site/about/guidelines.xhtml

Sense of coherence as a coping mechanism for women with anxiety living in deprivation: British population study

For peer review only

Olivia Remes[1], Nick Wainwright[2], Paul Surtees[1], Louise Lafortune[1], Kay-Tee Khaw[3], Carol Brayne[1]

[1]Department of Public Health and Primary Care, University of Cambridge, Forvie Site, Robinson Way, Cambridge, UK CB2 0SR

[2](Retired) Department of Public Health and Primary Care, University of Cambridge, 2 Worts' Causeway, Cambridge, UK CB1 8RN

[3]Department of Public Health and Primary Care, University of Cambridge, 2 Worts' Causeway, Cambridge, UK CB1 8RN

Figures: 1; Tables: 3; Appendix: 2

Correspondence to: Olivia Remes or260@medschl.cam.ac.uk, telephone: 0777674550

Abstract

OBJECTIVE

Many patients receiving medical treatment for anxiety relapse or do not improve. Research has therefore been turning to coping mechanisms as a way to decrease anxiety rates. Previously, we showed that living in a deprived area significantly increases the risk of anxiety in women, but not in men. The objective of this study is to assess whether sense of coherence (coping mechanism) buffers the influence of area deprivation on women's risk of generalized anxiety disorder using data from the European Prospective Investigation of Cancer-Norfolk.

DESIGN

Large, population study.

SETTING

UK population-based cohort.

PARTICIPANTS

30,445 people over the age of 40 were recruited through general practice registers in England. Of these, 20,919 completed a structured health and lifestyle questionnaire used to assess generalized anxiety disorder and sense of coherence. Area deprivation was measured using 1991 Census data, and sense of coherence and anxiety were examined in 1996-2000. 10,183 women had complete data on all covariates.

MAIN OUTCOME MEASURE

Past-year generalized anxiety disorder defined according to the Diagnostic and Statistical Manual of Mental Disorders, fourth edition.

RESULTS

In this study, 2.6% (260/10,183) of women had generalized anxiety disorder. In those with a strong sense of coherence, area deprivation was not significantly associated with anxiety (OR=1.29, 95% CI: 0.77, 2.17). However, among women with a weak sense of coherence, those living in deprived areas were almost twice as likely to have generalized anxiety disorder compared to those living in more affluent areas (OR=1.99, 95% CI: 1.37, 2.91).

CONCLUSION

The absolute number of women living in deprived conditions is large worldwide, and significant numbers are affected by generalized anxiety disorder. Sense of coherence moderates the association between area deprivation and anxiety in women; therefore, interventions targeting coping mechanisms may need to be considered for people with anxiety.

Key words: Anxiety, anxiety disorders, risk factors, gender

For peer review only

Article summary

Strengths and limitations of this study

- We used a large, population-based sample of middle- and older-aged adults and adjusted for a range of important confounders, such as, sociodemographic factors and medical history.

- We used a structured, self-reported questionnaire to assess presence of past-year GAD, and sense of coherence.

- We measured area deprivation and sense of coherence by using common, valid and theoretically-sound indices.

- Those who participated in this study were somewhat less deprived and healthier than individuals living in other parts of England; therefore, our results may not generalize to people living in extremely deprived circumstances.

For peer review only

Introduction

Generalized anxiety disorder (GAD)[1] is one of the most common anxiety disorders in the general population.[2-4] It is characterized by excessive and pervasive worry about a number of areas of life, and associated symptoms, such as, restlessness, irritability, muscle tension, sleep difficulties, and concentration problems.[1] If left untreated, this disorder can increase the risk for disability, impairment, and suicide.[2-5] Although treatment for anxiety exists in the form of psychotherapy and pharmacotherapy, very few people who need treatment actually receive it.[6] One of the reasons for this is that physicians under- and misdiagnose those affected, and few people experiencing symptoms seek help from the clinician.[7] Low rates of help-seeking is a result of low general awareness about the disorder and treatment options, and people perceiving their anxiety to be an intractable personality trait, rather than a condition that can be treated. These problems are further compounded by the fact that even after patients are treated, many relapse, while some do not experience improvement in symptoms.[7]

While it is not known what causes anxiety, most studies on risk have focused on individual-level determinants of anxiety disorders such as personal income, education and history of psychopathology.[8-11] However, research has shown that the environment can have a profound effect on mental health, over and above individual-level circumstances. The living context, such as, living in a deprived area, can have harmful effects for mental health independently of personal socio-economic status and lifestyle factors.[12,13] Women have been reported to be particularly affected by their context or the environment in which they are living.[14,15] Women living in poor areas seem to be disproportionately affected by mental disorders.[16, 17]. Previously, we showed that women living in deprivation had a

For peer review only

significantly higher risk of GAD, while this was not observed in men.[16] If women are living in an area with low socio-economic circumstances, they are more likely to be exposed to the stress and strain that arises from deprivation.[14] Exposure to stress can then increase the risk for central nervous system dysfunction and hypothalamic-pituitary-adrenal axis dysregulation, which may lead to the development of GAD.[18,19]

To reduce the risk of mental disorders among women exposed to disadvantage or adversity, coping skills need to be considered. In particular, sense of coherence (SOC), which is a way of viewing life as predictable, manageable, and meaningful, can lower the risk for poor health outcomes.[20,21] Also, SOC is a flexible and adaptive dispositional orientation which enables coping with stressful situations.[21,22]

Two systematic reviews[20,23] showed that SOC is linked to quality of life. A strong SOC is related to good physical and self-perceived health, and is negatively associated with anxiety, depression, and post-traumatic stress disorder (PTSD).[23] In the European-Prospective Investigation of Cancer-Norfolk (EPIC-Norfolk) study of over 18,000 people, a strong SOC contributed to a 20% reduction in all-cause mortality in adults.[24] SOC has also been shown to moderate the influence of disadvantage on mental health outcomes. In a study of people who had faced early childhood deprivation and trauma during the Holocaust, SOC moderated the association between early-life deprivation and posttraumatic stress in old age.[25] A strong SOC can therefore be a major coping resource for preserving health.

Previously[16], we have shown that women living in deprived areas were at increased risk for GAD. The stress of living in deprivation was harmful for women’s mental health, while

For peer review only

this association with deprivation was not apparent in men. For this reason, this study will focus on women. The objective of this study is to determine whether SOC moderates the link between area deprivation and GAD in women using a large, longitudinal, population cohort.

For peer review only

Methods

Study population

Data were drawn from the population-based European Prospective Investigation of Cancer (EPIC)-Norfolk, described in detail elsewhere[26]. Between 1993 and 1997, 30,445 participants ages 40 to 74 years living in Norwich and the surrounding towns and rural areas were identified through general practice age-sex registers (77,630 people were initially invited to join EPIC-Norfolk). At baseline (1993-97), 30,445 participants consented to join the study and completed a postal Health and Lifestyle (HLQ) questionnaire that captured information on sociodemographics, including gender, marital status, highest educational attainment, employment, and self-reported physician diagnoses of physical diseases. Using participants' postal codes, a measure of area deprivation was derived based on the 1991 Census.[27] Between 1993 and 2000, participants completed self-reported postal questionnaires provided they: 1) were still alive, 2) did not ask to be removed from the study's mailing list, and 3) had a valid mailing address.

All participants recruited through general-practice registers and who completed a baseline health questionnaire were eligible to be included in our study; those who completed a psychosocial questionnaire during follow-up were eligible to be included in our analysis.

Assessment of GAD – outcome

In 1996-2000, 20,919 men and women completed a Health and Life Experiences Questionnaire (HLEQ)[28] used to identify those meeting criteria for DSM-IV GAD. The primary outcome in this study was past-year GAD. The HLEQ captured the onset and offset

For peer review only

timings of episodes of GAD.[29] Past-year GAD consisted of at least one episode that had offset within 12 months of administration of the HLEQ. DSM-IV GAD was diagnosed if participants reported having uncontrollable, excessive worry for six months or longer on most days than not that resulted in disability or impairment. In addition, at least three of the following symptoms needed to have been present: restlessness, irritability, muscle tension, fatigue, trouble concentrating because of worry, mind going blank, trouble falling asleep, trouble staying asleep, and feeling keyed up or on edge. Of those who completed the HLEQ, 462 met criteria for past-year DSM-IV GAD.

Assessment of potential confounders

Covariates were chosen a priori based on previous literature (their links to anxiety[30-33] and deprivation[34]). The baseline HLQ was used to ascertain gender, education (highest level of education attained: no qualifications, educated to age 16 years, educated to age 18 years, or educated to degree level), marital status (single, married, widowed, separated, divorced), employment (yes, no), and self-reported physician diagnoses of major medical conditions (self-reported asthma, bronchitis, allergies, hay fever, stroke, heart attack, cancer, diabetes, thyroid conditions, arthritis). Social class (professionals, managerial and technical occupations, skilled workers divided into non-manual and manual, partly skilled workers and unskilled manual workers) was derived using the Computer-Assisted Standard Occupational Coding.[35]

The HLEQ was used to derive participant age, determine presence of lifetime MDD according to the DSM-IV, and disability measures based on the SF-36. To determine disability levels, we used the physical component summary score (PCS) of the Medical

For peer review only

Outcomes Study 36-Item Short Form (SF-36), a widely-used, validated self-assessment tool. Higher scores indicate better health.[36] PCS scores were dichotomized above and below the median.

Assessment of area deprivation – exposure

To examine area deprivation, we used the Townsend Index.[37,38] This index is a composite measure of four variables obtained from the 1991 Census: 1) percentage of economically active residents over age 16 who are unemployed, 2) percentage of households that do not possess a car, 3) percentage of private households that are not owner occupied, and 4) percentage of private households that are overcrowded (have more than 1 person per room). These variables were obtained at the level of the enumeration district. For each variable, Z scores were obtained by dividing the mean by the standard deviation (across enumeration districts in England). The Z-values of the four variables were added together to produce a Townsend index score. Positive values of the index indicate areas that are more deprived, while negative values indicate areas that are less deprived; 0 represents the national mean. The postal codes of participants were record linked to enumeration districts, and participants were considered to live in deprived areas depending on the Townsend index score assigned to their enumeration district.

For peer review only

Ascertainment of SOC

The HLEQ included a three-item SOC questionnaire[39] that assessed each of the SOC constructs. The following questions were used to assess each construct:

Comprehensibility:

Do you usually feel that the things that happen to you in your daily life are hard to understand?

Manageability:

Do you usually see a solution to problems and difficulties that other people find hopeless?

Meaningfulness:

Do you usually feel that your daily life is a source of personal satisfaction?

Participants were given the choice of responding to these questions with yes, usually; yes, sometimes; and no. Comprehensibility was reverse scored, and all items were then summed to provide a total SOC scale ranging from 0 to 6. Higher scores represent weaker SOC.

For peer review only

Statistical analysis

Characteristics of the participants were compared by GAD status. We used correlated data analysis to assess the association between individual- and area-level risk factors of GAD in women and men, separately. A population-average model was constructed, which accounted for the potential correlation introduced by the clustering of individuals within enumeration districts. To estimate the population-average effect of the risk factors of interest on past-year GAD, we used generalized estimating equations (GEE). As past-year GAD represents a binary outcome (yes/no) and the intra-cluster correlation is assumed to be equal, GEE with a logit link and an exchangeable correlation structure was used. Adjusted odds ratios (OR) and 95% confidence intervals based on robust standard errors were estimated. Standard multivariate logistic regression was also conducted and compared to the findings based on GEE.

Individual-level measures consisted of demographic, socio-economic status, and health variables, whereas the area-level measure was the Townsend index. Townsend index scores were used to create a dichotomous variable, with 0 as the cut-point (representing the national average).

SOC was split at the median (of 2) and participants below this cut-point were classified as weak on SOC, while those above this cut-point had a strong SOC. The interaction between area deprivation and SOC in women was assessed. After this, analyses were conducted separately for those with strong and weak levels of SOC. First, unadjusted effect estimates were determined. Next, models were constructed that adjusted for 1) age, educational attainment, marital status, social class, and employment; then for 2) age, educational

For peer review only

attainment, marital status, social class, employment, and lifetime MDD; and finally for 3) age, educational attainment, marital status, social class, employment, lifetime MDD, physical diseases, and disability level. Age was assessed as a categorical variable. Models were constructed for participants with complete measurements on all covariates. The brackets show the reference categories that were used for each categorical variable when it was entered in the models – age: young (<65) vs. old (>=65) [ref]; education: high [ref] vs. low; marital status: married [ref] vs. not married; social class: non-manual [ref] vs. manual; employed: no vs. yes [ref]; lifetime MDD: no [ref] vs. yes; deprivation: no [ref] vs. yes; prevalent physical disease: no [ref] vs. yes; disability level: low [ref] vs. high. These reference categories were based on the literature. Choosing other groupings for the potential confounders would not have changed the results. It was not possible to group the GAD variable otherwise, and area deprivation was analysed in accordance with the literature.

To arrive at the study size, we went through the following steps: of the 30,445 who completed the baseline HLQ, we retained those participants (both men and women) who completed the HLEQ (20,919), and of these, we kept only women with complete data on all covariates (10,183). (Figure 1)

Patient involvement

There were no patients involved in the development of the research question and outcome measures, the design of the study, or the recruitment to and conduct of the study.

For peer review only

Results

77,630 people from general practices in Norfolk were invited to take part in the study, and of these, 30,445 consented.[26] The characteristics of responders versus non-responders are compared in Appendix 1; compared to non-responders, those who took part consisted of slightly more women and slightly younger participants. Of the 30,445 people recruited at baseline, 20,919 completed the HLEQ during follow-up.[26,29] Of those who completed the HLEQ, 10,183 women were retained for analysis in this study, because they had complete data on all covariates. The number of missing observations for each covariate was: 1 for age, 7 for education, 23 for marital status, 303 for social class, 35 for employment, 46 for Townsend index, 883 for disability, 321 for MDD, and 300 for GAD. Participants were assessed between 1993 and 2000 (followed for 7 years) (Figure 1).

In 1996-00, GAD was present in 260 out of 10,183 (2.6%) women. Table 1 shows sociodemographic, medical history, and lifestyle characteristics for women with a weak and strong SOC.

For peer review only

Table 1: Distribution of characteristics for women (n=10,183) with weak and strong SOC who completed the HLEQ questionnaire in the EPIC-Norfolk cohort

	Weak SOC		Strong SOC	
**Characteristic**	**Number with characteristic**	**Percentage and number with past-year GAD**	**Number with characteristic**	**Percentage and number with past-year GAD**
**Socio-demographics**				
**Age (years)**				
<65	1995	6.8 (136)	4732	1.6 (78)
>=65	996	2.7 (27)	2460	0.8 (19)
**Education**[‡]				
Low	1358	4.6 (62)	2619	0.8 (21)
High	1633	6.2 (101)	4573	1.7 (76)
**Marital status**				
Married	2060	5.5 (113)	5590	1.2 (69)
Not married[*]	931	5.4 (50)	1602	1.7 (28)
**Social class**[¥]				
Manual	1261	4.9 (62)	2508	1.1 (27)
Non-manual	1730	5.8 (101)	4684	1.5 (70)
**Employed**				
Yes	1178	5.6 (66)	2852	1.4 (40)
No	1813	5.4 (97)	4340	1.3 (57)
**Townsend index**				
**Deprivation**				
Yes (>0)	534	8.4 (45)	1083	1.8 (19)
No (<=0)	2457	4.8 (118)	6109	1.3 (78)
**Health status**				
**Prevalent physical disease**				
Yes[+]	1717	6.2 (107)	3922	1.8 (70)
No	1274	4.4 (56)	3270	0.8 (27)
**Disability level**				
High[¶]	1717	6.3 (107)	3493	1.8 (64)
Low	1274	4.4 (56)	3699	0.9 (33)
**Lifetime MDD**				
Yes	737	13.8 (102)	1180	5.4 (64)
No	2254	2.7 (61)	6012	0.5 (33)

[‡] High education: O-level, A-level, degree; low education: refers to no education
[*] Single divorced, separated, widowed
[¥] Manual: skilled manual, semi-skilled, non-skilled; non-manual: professionals, managerial, skilled non-manual
[+] Prevalent physical disease: respiratory disease (asthma and bronchitis), allergies (allergies and hay fever), stroke, heart attack, cancer, diabetes, thyroid conditions, arthritis
[¶] Below the median PCS value of 50.6

For peer review only

Among women with a weak SOC, those who also had GAD were more likely to be younger, have high educational attainment, non-manual social class, live in areas of high deprivation, and have had pre-existing health conditions or show prevalent physical disease, high disability, and lifetime MDD. In the group with strong SOC, similar patterns were found as for those with anxiety.

During the 6-year follow-up period, there were a total of 260 GAD cases in women. A weak SOC was found in 2991 women, while a strong SOC was present in 7192 women. When the interaction between area deprivation and SOC was assessed, the p-value was 0.226. When area deprivation was regressed against SOC in a fully-adjusted model, the p-value was 0.372; and when area deprivation and SOC were introduced in a fully-adjusted model with GAD as the outcome, the p-values for these explanatory variables were 0.0005 and <0.0001, respectively.

Tables 2 and 3 show the unadjusted and adjusted odds ratios (Models A-C) associated with GAD in those with a weak and strong SOC, respectively.

For peer review only

Table 2: Odds ratios for women with a weak SOC who completed the HLEQ questionnaire in 1996-00 (women with weak SOC sample size=2,991)

	Odds ratios and 95% CI				
**Characteristic**	**Unadjusted**	**Model A[1]**	**Model B[2]**	**Model C[3]**	**P-value for Model C**
**Socio-demographics**					
**Age**					
<65	2.63 (1.72, 4.00)	3.35 (2.10, 5.34)	2.49 (1.54, 4.02)	2.67 (1.65, 4.32)	<0.0001
>=65	1.00	1.00	1.00	1.00	
**Education[‡]**					
Low	0.73 (0.52, 1.00)	0.77 (0.55, 1.08)	0.83 (0.58, 1.17)	0.83 (0.58, 1.17)	0.287
High	1.00	1.00	1.00	1.00	
**Marital status**					
Married	1.00	1.00	1.00	1.00	
Not married[*]	0.98 (0.69, 1.38)	1.06 (0.74, 1.51)	0.86 (0.59, 1.25)	0.85 (0.59, 1.23)	0.392
**Social class[¥]**					
Manual	0.83 (0.60, 1.15)	0.81 (0.58, 1.15)	0.83 (0.59, 1.18)	0.81 (0.57, 1.15)	0.231
Non-manual	1.00	1.00	1.00	1.00	
**Employed**					
Yes	1.00	1.00	1.00	1.00	
No	0.95 (0.69, 1.31)	1.55 (1.09, 2.19)	1.42 (0.99, 2.04)	1.33 (0.92, 1.91)	0.126
**Townsend index**					
**Deprivation**					
Yes (>0)	1.82 (1.28, 2.61)	2.05 (1.43, 2.94)	1.97 (1.35, 2.88)	1.99 (1.37, 2.91)	0.0004
No (<=0)	1.00	1.00	1.00	1.00	
**Health status**					
**Lifetime MDD**					
Yes	5.77 (4.15, 8.03)		5.20 (3.68, 7.34)	5.06 (3.58, 7.15)	<0.0001
No	1.00		1.00	1.00	
**Prevalent physical disease[+]**					
Yes	1.36 (0.98, 1.88)			1.20 (0.84, 1.70)	0.316
No	1.00			1.00	
**Disability level**					
High[¶]	1.45 (1.04, 2.01)			1.50 (1.04, 2.15)	0.030
Low	1.00			1.00	

1. Adjusted for age, SES (education, marital status, social class, employment)
2. Adjusted for age, SES, lifetime MDD
3. Adjusted for age, SES, lifetime MDD, prevalent physical disease and disability

[‡] High education: O-level, A-level, degree; low education: refers to no education

For peer review only

[*] Not married: single, divorced, separated, widowed
[¥] Manual: skilled manual, semi-skilled, non-skilled; non-manual: professionals, managerial, skilled non-manual
[+] Prevalent physical disease: respiratory disease (asthma, bronchitis), allergies (allergies, hay fever), stroke, heart attack, cancer, diabetes, thyroid conditions, arthritis
[¶] Below the median PCS value of 50.6

For peer review only

Table 3: Odds ratios for women with a strong SOC who completed the HLEQ questionnaire in 1996-00 (women with a strong SOC sample size =7,192)

	Odds ratios and 95% CI				
**Characteristic**	**Unadjusted**	**Model A[1]**	**Model B[2]**	**Model C[3]**	**P-value for Model C**
**Socio-demographics**					
**Age**					
<65	2.15 (1.30, 3.56)	2.58 (1.48, 4.50)	1.89 (1.06, 3.38)	2.13 (1.18, 3.85)	0.0118
>=65	1.00	1.00	1.00	1.00	
**Education[‡]**					
Low	0.48 (0.29, 0.78)	0.54 (0.33, 0.89)	0.59 (0.36, 0.98)	0.59 (0.35, 1.00)	0.0483
High	1.00	1.00	1.00	1.00	
**Marital status**					
Married	1.00	1.00	1.00	1.00	
Not married	1.42 (0.91, 2.22)	1.56 (0.99, 2.47)	1.25 (0.78, 2.01)	1.22 (0.76, 1.96)	0.4131
**Social class[¥]**					
Manual	0.72 (0.46, 1.12)	0.84 (0.53, 1.34)	0.86 (0.53, 1.39)	0.83 (0.52, 1.35)	0.4592
Non-manual	1.00	1.00	1.00	1.00	
**Employed**					
Yes	1.00	1.00	1.00	1.00	
No	0.94 (0.62, 1.41)	1.46 (0.94, 2.26)	1.44 (0.92, 2.25)	1.25 (0.79, 1.97)	0.3461
**Townsend index**					
**Deprivation**					
Yes (>0)	1.38 (0.83, 2.29)	1.43 (0.86, 2.39)	1.32 (0.79, 2.21)	1.29 (0.77, 2.17)	0.3366
No (<=0)	1.00	1.00	1.00	1.00	
**Health status**					
**Life-time MDD**					
Yes	10.39 (6.79, 15.89)		9.32 (6.05, 14.35)	8.58 (5.53, 13.31)	<0.0001
No	1.00		1.00	1.00	
**Prevalent physical disease[+]**					
Yes	2.18 (1.40, 3.41)			1.72 (1.10, 2.71)	0.0185
No	1.00			1.00	
**Disability level**					
High[¶]	2.07 (1.36, 3.16)			1.92 (1.21, 3.05)	0.0059
Low	1.00			1.00	

1. Adjusted for age, SES (education, marital status, social class, employment)
2. Adjusted for age, SES, lifetime MDD
3. Adjusted for age, SES, lifetime MDD, prevalent physical disease and disability

[‡] High education: O-level, A-level, degree; low education: refers to no education

[*] Not married: single, divorced, separated, widowed

[¥] Manual: skilled manual, semi-skilled, non-skilled; non-manual: professionals, managerial, skilled non-manual

For peer review only

[+] Prevalent physical disease: respiratory disease (asthma, bronchitis), allergies (allergies, hay fever), stroke, heart attack, cancer, diabetes, thyroid conditions, arthritis

[¶] Below the median PCS value of 50.6

For peer review only

Analyses that adjusted for age, education, marital status, social class, and employment status showed that area deprivation was significantly associated with increased risk for GAD in women with a weak SOC (OR=2.05, 95% CI: 1.43, 2.94) (table 2), but area deprivation was not significantly associated with anxiety in those with strong SOC (OR=1.43, 95% CI: 0.86, 2.39) (table 3). In women with a weak SOC (table 2), further adjustment for lifetime MDD slightly attenuated the effect estimate, though the association between area deprivation and anxiety remained highly significant (OR=1.97, 95%: 1.35, 2.88). When prevalent physical disease and disability level were added to the final model, the effect estimate remained almost unchanged compared to the previous model; among women with poor coping skills, those living in deprived areas had a 99% higher likelihood of having anxiety than women living in less deprived areas (OR=1.99, 95% CI: 1.37, 2.91). For women with a strong SOC (table 3), area deprivation was associated with a small increased risk of having GAD in progressively adjusted models; however, none of the effect estimates reached statistical significance. In the fully-adjusted model, women with a strong SOC and living in deprivation had a 29% higher chance of having GAD compared to women living in less deprived areas, but this did not reach statistical significance (OR=1.29, 95% CI: 0.77, 2.17).

We had similar findings when logistic regression was used in these models instead of GEE, suggesting that the intra-class correlation is negligible (weak SOC: OR=1.29 [95% CI: 0.77, 2.18] and strong SOC: OR=1.99 [95% CI: 1.36, 2.93]).

We carried out multiple imputations for missing data (Appendix 2); the effect estimate became slightly stronger for women with a weak SOC and living in deprivation (OR=2.21,

For peer review only

95% CI: 1.56, 3.13), and the association between deprivation and anxiety become even weaker for women with a strong SOC (OR=1.12, 95% CI: 0.69, 1.88).

For peer review only

Discussion

In this large, population-based study, we found that area deprivation significantly increased the risk for generalized anxiety disorder (GAD) in women, but particularly in those with poor coping skills. Coping skills or sense of coherence (SOC) appeared to moderate the association between area deprivation and anxiety. SOC was based on a simplified 3-item measure, with modest internal reliability (Cronbach's alpha=0.35)[22], and this variable was dichotomized. Although it may be useful to additionally employ a continuous SOC measure, we dichotomized this variable because previous literature had done so as well.[24]

Women living in deprivation and with poor coping or a weak SOC were at a particularly high risk for having anxiety after controlling for important confounders. Although women with a strong SOC showed a slight increased risk of anxiety if living in disadvantaged circumstances, the association between area deprivation and GAD was statistically non-significant in women who were able to cope well and the effect estimate was much smaller than that of the former group (women with poor coping). A statistically significant association between area deprivation and GAD persisted in women with a weak SOC after adjustment for age, marital status, education level, social class, employment status, major depressive disorder, chronic physical diseases, and disability. In contrast, having a strong SOC seemed to be protective for women living in deprived areas. Having a strong SOC rendered the association between area deprivation and anxiety statistically non-significant.

Although the interaction between area deprivation and SOC was not statistically significant, the effect estimates do suggest that there are differences between women with low and high SOC – nevertheless, these differences are rather small. Our study sheds light on the

For peer review only

potential importance of SOC when it comes to mitigating the risks of anxiety. Future research should replicate our study with a larger number of anxiety cases, perhaps by measuring ‘total’ or ‘any’ anxiety rather than individual disorders, such as GAD.

Deprived areas are often associated with low social integration and poor social control. Emile Durkheim showed that low social integration can lead to a sense of meaninglessness among individuals, and this can give rise to poor mental health and suicide.[40] SOC is a way of viewing life as meaningful and comprehensible, and our study shows that SOC can moderate the association between area deprivation and GAD in women.

For peer review only

Strengths and limitations of this study, and future research

This is the largest, population-based study of the association between area deprivation and GAD in women, and to determine whether coping resources or SOC moderates the association between area deprivation and anxiety. We had access to a large sample of over 10,000 women living in the community. We used a clinically relevant measure of anxiety, and GAD was defined according to the Diagnostic and Statistical Manual of Mental Disorders, fourth version (DSM-IV). Although GAD affects a substantial number of people, even more experience subthreshold cases of anxiety disorders. Subthreshold cases have also been associated with impairment and disability; therefore, future research should assess associations with subclinical anxiety.

We used detailed health and lifestyle questionnaires to extract information on demographics, social class, and major chronic physical diseases, and controlled for these factors in our analyses. We used a validated and reliable measure of disability, which we adjusted for in our models. We had a large list of self-reported physician diagnoses that we used to establish medical histories for participants, though three issues might arise with this approach. First, the residual effect of diseases not captured by our study but that are associated with area deprivation and anxiety, may be present. Second, medical diagnoses were not verified by clinicians, leading to possible misclassification. Third, past illness may have been under-reported, leading to misclassification bias and attenuation of effect estimates. We may have overadjusted our models with the inclusion of disability, because this might be part of the expression of psychiatric illness. This may have reduced effect estimates. Our objective was to assess the links between deprivation, SOC, and anxiety in women. Although it was out of scope for the present study, we were unable to examine the

For peer review only

same objectives in men: there were very few men with a strong SOC living in deprivation and with GAD. Therefore, analyses in this sub-group would not have been robust. Future studies should undertake this assessment. It should also be mentioned that the internal consistency of the three-items SOC scale, as measured by Chronbach's alpha, was 0.35.[22] While the internal consistency of the shorter 3-item measure was low in this sample, this is likely to be partially due to the small number of scale items. Also, the original developers of the scale reported satisfactory short-term test-retest reliability and validity for the 3-item measure.[22,41] Despite this, it was a limitation that we did not have a longer measure with higher reliability, such as the SOC-13 or SOC-29.[41] The SOC was dichotomized – it may be useful to additionally use a continuous measure of SOC, but we dichotomized it because research on coping had done so as well.[24]

At baseline, people who consented to take part in EPIC-Norfolk agreed to fill out detailed health and lifestyle questionnaires over the duration of the study period; therefore, healthy volunteer effect may have biased our findings. Participants in EPIC-Norfolk tend to be somewhat healthier and more affluent than the general population, therefore, results from this study cannot be generalized to extremely deprived areas. If the most deprived areas would have been included, we would expect the association between area deprivation and anxiety to be even stronger in women with a weak SOC. Also, when comparing the demographic characteristics of responders versus non-responders (Appendix 1), we found that participants were slightly younger and slightly more women than men consented.

Also, it may be that participants with poorer mental health may have moved to more deprived neighbourhoods; however, reverse causality seems unlikely as an explanation for

For peer review only

our findings. In addition, deprivation was measured before anxiety in this study; however, SOC was examined at the same time point as GAD, rendering this study cross-sectional.

Non-participation in our study may have contributed to non-differential misclassification and attenuation of effect estimates. Although our study is observational in nature and cannot confirm causality between area deprivation and GAD in women with a strong and weak SOC, a rigorous analysis based on observational data is a reasonable way of examining this relationship. When we conducted multiple imputations, the effect estimate for women with a weak SOC became even greater, and among women with a strong SOC, it attenuated towards the null. Our study provides a valuable step forward and is the first to shed light on the importance of coping in people with GAD living in disadvantaged circumstances.

Comparison with other studies

This is the largest, population-based study to consider the association between area deprivation and GAD in women, and to determine whether SOC moderates this association. Most of the literature on coping and SOC is limited. Most studies have small sample sizes, and measure people's coping abilities in relation to feelings of stress, history of stressful life events, or exposure to stressful circumstances, such as, wars. There is a paucity of research examining the living context, such as, area deprivation, and no studies have assessed whether the link between area-level circumstances and anxiety disorders can be moderated by coping mechanisms. The literature on coping uses highly select samples; therefore, results cannot be generalized to the larger population. Also, incomplete adjustment of covariates makes it difficult to determine whether findings from these studies are not better explained by the residual effect of other factors that have not been accounted for, such as,

For peer review only

lifestyle and personal socio-economic circumstances. Across studies, there is large heterogeneity in the definitions used to define coping, with many focusing on factors, such as, hardiness, optimism, and negative emotions, rather than SOC. In sum, it is difficult to understand the links between the living context, coping abilities, and mental health from the literature; however, the studies that have been conducted are a good starting point.

A UK study of over 3000 people [42] showed that SOC was linked to self-rated health; however, the moderating effect of coping was not assessed. Research on people living in Negev communities in Israel showed that those exposed to trauma and severe stress-provoking situations, but who had a strong SOC, were least likely to develop stress.[43] In a study of French adults [44], SOC buffered the effect of adversity on psychological well-being. In another study of Holocaust survivors [25], SOC moderated the association between early childhood deprivation and posttraumatic stress in old age. Both of these latter studies, however, were small, failed to adjust for important confounders, such as sociodemographic factors and disability, and did not examine individual psychiatric disorders diagnosed according to valid and reliable criteria, such as, the DSM. In the study on child Holocaust survivors [25], exposure to trauma was measured in early life, while posttraumatic stress in old age. Since participants were required to report traumas experienced in childhood, this might have led to recall bias. Our study expands on previous research and is the first to investigate the moderating effect of coping skills (SOC) on the risk of developing GAD in women living in deprived circumstances.

For peer review only

Mechanism of effect

Living in a deprived area can increase anxiety in women because of biological and social factors.[16] The stress of living in deprivation can increase the risk for inflammation and HPA-axis dysregulation, which can lead to GAD.[18,19,45] This, combined with the multiple roles that women are increasingly taking on (income earner, child-bearer, and carer of elderly relatives)[45], means that coping is particularly relevant for women living in disadvantaged circumstances. A strong SOC is linked to high quality of life, and good physical and mental health.[20,23] Our study shows that SOC can buffer the effect of area deprivation on risk of anxiety.

Implications

The absolute number of people living in deprived conditions is large worldwide, and significant numbers will have been affected by GAD.[46] For the first time, we show that SOC moderates the association between area deprivation and anxiety in women. Future research should replicate our analysis using larger samples and determine the specific components of SOC that attenuate the effect of deprivation on mental health. Interventions can then be developed to target components of SOC to increase people's coping resources. Treatment for GAD exists, with psychotherapy and pharmacotherapy being commonly prescribed. However, success rates are fairly low, patients relapse, and some fail to experience any symptom improvement. Costs to the health care system related to anxiety are substantial. Therefore, targeting people's coping resources could represent another option for people with anxiety, including those who do not experience symptom improvement following commonly-prescribed therapies. Targeting SOC could also represent a better option for people who have faced extreme circumstances and adversity,

For peer review only

and who may have difficultly dealing with the traumas directly as during psychotherapy. Interventions should take these findings into account, and mental health policy should also consider improving living environments to decrease the burden of anxiety in women.

For peer review only

Acknowledgements: OR received a PhD studentship from the National Institute for Health Research.

Competing interest: All authors have completed the ICMJE uniform disclosure form at www.icmje.org/coi_disclosure.pdf and declare: no support from any organisation for the submitted work; no financial relationships with any organizations that might have an interest in the submitted work in the previous three years; no other relationships or activities that could appear to have influenced the submitted work.

Funding: This work was supported by the Medical Research Council UK (grant number SP2024-0201 and SP2024-0204) and Cancer Research UK (grant number G9502233).

Author contributions: OR (corresponding author) had the idea for and conducted the analysis, and wrote the article. CB critically reviewed drafts of the manuscript and provided input into the analysis, KK edited versions of the paper; PS and NW provided feedback into the analysis and reviewed the final draft of the paper. OR, CB, KK, LL, PS, and NW contributed to the interpretation of data for the work, agreed to be accountable for all aspects of the work, gave final approval of the version to be published, and made substantial contributions to the analysis and interpretation of data. OR, CB, KK, LL, PS, and NW have seen and approved the final version. OR, CB, KK, LL, PS, and NW had full access to all the data in the study and take responsibility for the integrity of the data and the accuracy of the data analysis. OR acts as guarantor of the study.

For peer review only

Transparency declaration: OR affirms that the manuscript is an honest, accurate, and transparent account of the study being reported; that no important aspects of the study have been omitted; and that any discrepancies from the study as planned have been explained.

Role of study sponsors and statement of independence: The funding sources had no role in the design and conduct of the study; collection, management, analysis, and interpretation of the data; and preparation, review, or approval of the manuscript.

Ethical approval: The study has ethics committee approval from Norfolk Ethics Committee (Rec Ref: 98CN01) and all participants gave informed consent.

Data sharing: No additional data available. Original dataset requests should be sent to the corresponding author. Please contact O Remes at or260@medschl.cam.ac.uk for questions about the statistical code.

Copyright: "The Corresponding Author has the right to grant on behalf of all authors and does grant on behalf of all authors, a worldwide licence to the Publishers and its licensees in perpetuity, in all forms, formats and media (whether known now or created in the future), to i) publish, reproduce, distribute, display and store the Contribution, ii) translate the Contribution into other languages, create adaptations, reprints, include within collections and create summaries, extracts and/or, abstracts of the Contribution, iii) create any other

For peer review only

derivative work(s) based on the Contribution, iv) to exploit all subsidiary rights in the Contribution, v) the inclusion of electronic links from the Contribution to third party material where-ever it may be located; and, vi) licence any third party to do any or all of the above."

For peer review only

References

1. American Psychiatric Association. (1994). *Diagnostic and Statistical Manual of Mental Disorders* (4th edn). Washington, DC: APA.
2. Kessler R, Keller M, Wittchen H. The epidemiology of generalized anxiety disorder. *Psychiatr Clin North Am* 2001;24:19–39, doi: http://dx.doi.org/10.1016/S0193-953X(05)70204-5 [published Online First 29 June 2005].
3. Stein MB, Sareen J. Generalized anxiety disorder. *New Engl J Med* 2015;373:2059–2068, doi: 10.1056/NEJMcp1502514 [published Online First 19 November 2015].
4. Hoge E, Ivkovic A, Fricchione G. Generalized anxiety disorder: diagnosis and treatment. *BMJ* 2012;345,e7500, doi: http://dx.doi.org/10.1136/bmj.e7500 [published Online First 27 November 2012].
5. Nepon J, Belik S, Bolton J, Sareen J. The relationship between anxiety disorders and suicide attempts: findings from the National Epidemiologic Survey on Alcohol and Related Conditions. *Depress Anx* 2010;27:791–798, doi: 10.1002/da.20674 [published Online First 9 March 2010].
6. Revicki DA, Travers K, Wyrwich KW. Humanistic and economic burden of generalized anxiety disorder in North America and Europe. *J Affect Disord* 2012;140:103–12.
7. Simpson HB, Neria Y, Lewis-Fernandez R, Schneier F. Anxiety disorders: theory, research and clinical perspectives. Cambridge University Press, 2010.
8. Grant BF, Hasin DS, Stinson FS, et al. Prevalence, correlates, co-morbidity, and comparative disability of DSM-IV generalized anxiety disorder in the USA: results from the National Epidemiologic Survey on Alcohol and Related Conditions. *Psychol Med* 2005;35:1747, doi: 10.1017/S0033291705006069 [published Online First 5 October 2005].

For peer review only

9. The NHS Information Centre for health and social care, Leeds. Adult psychiatric morbidity in England, 2007 Results of a household survey. http://www.esds.ac.uk/doc/6379/mrdoc/pdf/6379research_report.pdf (accessed 29 July 2014).

10. Fryers T, Melzer D, Jenkins R. Social inequalities and the common mental disorders: a systematic review of the evidence. *Soc Psychiatry Psychiatr Epidemiol* 2003;38:229–237, doi: 10.1007/s00127-003-0627-2.

11. Zhang X, Norton J, Carriere I, Ritchie K, Chaudieu I, Ancelin ML. Risk factors for late-onset generalized anxiety disorder: results from a 12-year prospective cohort (the ESPRIT study). *Transl Psychiatry* 2015;5:e536, doi: 10.1038/tp.2015.31 [published Online First 31 March 2015].

12. Subramanian S, Kawachi I. Income inequality and health: what have we learned so far?. *Epidemiol Rev* 2004;26:78–91, doi: 10.1093/epirev/mxh003.

13. Pickett KE, Wilkinson RG. Income inequality and health: a causal review. *Soc Sci Med* 2014;128:316–326, doi: 10.1016/j.socscimed.2014.12.031 [published Online First 30 December 2014].

14. Pattyn E, Van Praag L, Verhaeghe M, Levecque K, Bracke P. The association between residential area characteristics and mental health outcomes among men and women in Belgium. *Arch Public Health* 2011;69:3, doi: 10.1186/0778-7367-69-3 [published Online First 24 October 2011].

15. Stafford M, Cummins S, Macintyre S, Ellaway A, Marmot M. Gender differences in the associations between health and neighbourhood environment. *Soc Sci Med* 2005;60:1681–1692, doi: 10.1016/j.socscimed.2004.08.028 [published Online First 5 November 2004].

For peer review only

16. Remes O, Wainwright N, Surtees P, Lafortune L, Khaw K, Brayne C. Sex differences in the association between area deprivation and generalized anxiety disorder: British population study. *BMJ Open* 2017 (in press).

17. Morrissey K. Gender differences in the association between common mental disorders and regional deprivation in Ireland. *Prof Geogr* 2015;68:129-137 doi: 10.1080/00330124.2015.1054020 [published Online First 24 July 2015].

18. Hoehn-Saric R, McLeod D, Funderburk F, Kowalski P. Somatic symptoms and physiologic responses in generalized anxiety disorder and panic disorder. *Arch Gen Psychiatry* 2004;61:913, doi: 10.1001/archpsyc.61.9.913.

19. Chaudieu I, Beluche I, Norton J, Boulenger JP, Ritchie K, Angelin ML. Abnormal reactions to environmental stress in elderly persons with anxiety disorders: evidence from a population study of diurnal cortisol changes. *J Affect Disord* 2008;106:307–313, doi: 10.1016/j.jad.2007.07.025 [published Online First 28 August 2007].

20. Eriksson M, Lindstrom B. Antonovsky's sense of coherence scale and its relation with quality of life: a systematic review. *J Epidemiol Community Health* 2007;61:938-944, doi: 10.1136/jech.2006.056028.

21. Antonovsky A. Unravelling the mystery of health. How people manage stress and stay well. San Francisco, CA: Lossey-Bass 1987.

22. Surtees PG, Wainwright NW, Khaw KT. Resilience, misfortune, and mortality: evidence that sense of coherence is a marker of social stress adaptive capacity. *J Psychosom Res* 2006;61:221-227, doi: 10.1016/j.jpsychores.2006.02.014.

23. Eriksson M, Lindstrom B. Antonovsky's sense of coherence scale and the relation with health: a systematic review. *J Epidemiol Community Health* 2006;60:376-381, doi: 10.1136/jech.2005.041616.

For peer review only

24. Wainwright NWJ, Surtees PG, Welch AA, Luben RN, Khaw K-T, Bingham SA. Sense of coherence, lifestyle choices and mortality. J Epidemiol Community Health 2008;62:829-831 doi: http://dx.doi.org/10.1136/jech.2007.066464.

25. van der Hal-van Raalte EAM, van IJzendoorn MH, Bakermans-Kranenburg MJ. Sense of coherence moderates late effects of early childhood holocaust exposure. J Clin Psychol 2008;64:1352-67. doi: 10.1002/jclp.20528. [published Online First 24 October 2008].

26. Hayat SA, Luben R, Keevil VL, et al. Cohort profile: a prospective cohort study of objective physical and cognitive capability and visual health in an ageing population of men and women in Norfolk (EPIC-Norfolk 3). *Int J Epidemiol* 2013;1-10 [published Online First 14 June 2013].

27. Office for National Statistics, UK. Census data 1801 1991. https://www.ons.gov.uk/census/2011census/2011censusdata/censusdata18011991 (accessed 20 Apr 2015).

28. Ishihara-Paul L, Wainwright NW, Khaw KT, et al. Prospective association between emotional health and clinical evidence of Parkinson's disease. *Eur J Neurol* 2008;15:1148-54, doi: 10.1111/j.1468-1331.2008.02299.

29. Surtees P, Wainwright N, Khaw K, Day N. Functional health status, chronic medical conditions and disorders of mood. *Br J Psychiatry* 2003;183:299–303, doi: 10.1192/bjp.183.4.299 [published Online First 30 September 2003].

30. Remes O, Brayne C, Van der Linde R, Lafortune L. A systematic review of reviews on the prevalence of anxiety disorders in adult populations. *Brain Behav* 2016;6:e00497 doi: 10.1002/brb3.497 [published Online First 5 June 2016].

For peer review only

For peer review only - http://bmjopen.bmj.com/site/about/guidelines.xhtml

31. Weisberg RB. Overview of generalized anxiety disorder: epidemiology, presentation, and course. *J Clin Psychiatry* 2009;70:4-9. doi: 10.4088/JCP.s.7002.01.

32. Wittchen HU. Generalized anxiety disorder: prevalence, burden, and cost to society. *Depress Anxiety* 2002;16:162-71 doi: 10.1002/da.10065 [published Online First 18 December 2002].

33. Culpepper L. Generalized anxiety disorder and medical illness. J Clin Psychiatry 2009;70:20-4.

34. Lakshman R, McConville A, How S, Flowers J, Wareham N, Cosford P. Association between area level socio-economic deprivation and a cluster of behavioural risk factors: Cross sectional, population based study. *J Public Health (Oxf)* 2011;33:234-245. doi: 10.1093/pubmed/fdq072 [published Online First 29 September 2010].

35. Elias P, Halstead K, Prandy K. CASOC: Computer-assisted standard occupational coding. London, England: HMSO 1993.

36. Ware JE, Snow K, Kosinski M, Gandek B. New England Medical Center Hospital. Health Institute. SF-36 Health Survey: Manual and Interpretation Guide. The Health Institute; Boston, MA: New England Medical Center 1993.

37. National Centre for Research Methods. Townsend deprivation index. http://www.restore.ac.uk/geo-refer/36229dtuks00y19810000.php (accessed 15 April 2014).

38. Shohaimi S, Luben R, Wareham N, et al. Residential area deprivation predicts smoking habit independently of individual educational level and occupational social class. A cross sectional study in the Norfolk cohort of the European Investigation into Cancer (EPIC-Norfolk). *J Epidemiol Community Health* 2003;57:270–276, doi: 10.1136/jech.57.4.270.

For peer review only

39. Surtees PG, Wainwright NWJ, Luben R, Khaw K-T, Day NE. Mastery, sense of coherence, and mortality: evidence of independent associations from the EPIC-Norfolk prospective cohort study. *Health Psychol* 2006;25:102-10, doi: 10.1037/0278-6133.25.1.102.

40. Durkheim E. Suicide – a study in sociology. Simon and Schuster 2010.

41. Lundberg O, Peck MN. A simplified way of measuring sense of coherence: experiences from a population survey in Sweden. *Eur J Pub Health*; 1995;5:56-59, doi: https://doi.org/10.1093/eurpub/5.1.56.

42. Walsh D, McCartney G, McCullough S, Buchanan D, Jones R. Comparing Antonovsky's sense of coherence scale across three UK post-industrial cities. BMJ Open 2014;4:e005792, doi: http://dx.doi.org/10.1136/bmjopen-2014-005792.

43. Braun-Lewensohn O, Sagy S. Community resilience and sense of coherence as protective factors in explaining stress reactions: comparing cities and rural communities during missiles attacks. *Community Mental Health J* 2014;50:229-234, doi: 10.1007/s10597-013-9623-5 [published Online First 9 June 2013].

44. Gana K. Is sense of coherence a mediator between adversity and psychological wellbeing in adults? *Stress and Health* 2001;17:77-83, doi: 10.1002/smi.882 [published Online First 26 February 2001].

45. Remes O, L Lafortune, K-T Khaw, C Brayne. Sex and gender in psychiatry. Lancet Psychiatry 2017;4:e1, doi: http://dx.doi.org/10.1016/S2215-0366(16)30445-X.

46. Whiteford HA, Degenhardt L, Rehm J, et al. Global burden of disease attributable to mental and substance use disorders: findings from the Global Burden of Disease Study 2010. *Lancet* 2013;9904:1575-1586 doi: http://dx.doi.org/10.1016/S0140-6736(13)61611-6 [published Online First 29 August 2013].

For peer review only

Figure 1 - Flowchart of EPIC-Norfolk cohort

This is a flowchart showing the number of participants at each study stage: the total number who completed the psychosocial HLEQ in the EPIC-Norfolk study, the number of women who filled out the HLEQ, and with complete data on all covariates. The EPIC-Norfolk study consists of middle-aged and older British people.

For peer review only

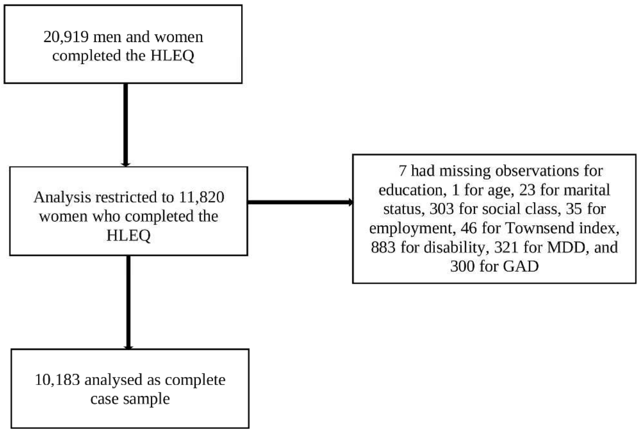

Flowchart showing the number of participants at each study stage: the total number who completed the psychosocial HLEQ in the EPIC-Norfolk study, the number of women who filled out the HLEQ, and with complete data on all covariates. The EPIC-Norfolk study consists of middle-aged and older British people.

Appendix 1: Characteristics of participants who consented (n=30,445) and refused (n=43,452) to take part in the EPIC-Norfolk cohort study

Percentage (number)		
**Characteristic**	**Consented**	**Did not consent**
**Age**		
<50	27.5 (8366)	33.7 (14647)
50-60	30.3 (9230)	29.5 (12819)
60-70	32.5 (9879)	27.4 (11898)
>=70	9.8 (2970)	9.4 (4088)
**Gender**		
Female	55.0 (16744)	49.0 (21296)
Male	45.0 (13701)	51.0 (22156)

For peer review only

Appendix 2 – Supplementary Material

We imputed missing data separately for women with a weak and strong sense of coherence (SOC). Based on the literature, we identified 12 potential auxiliary variables; however, we retained eight variables that were correlated with the variables in our model and were good predictors of the missing status. Our imputation model included all variables in the analysis model and the auxiliary variables.

To retain as much information as possible, we conducted the imputations on non-transformed data-the original variables in our dataset. We imputed data using the fully conditional specification, and specified a linear regression model for continuous data that were normally distributed; predictive mean matching for continuous data that were not normally distributed; and logistic regression for categorical variables. Variable estimates were subsequently averaged from 5 imputed datasets using Rubin's rules (we transformed the data before running the analytic model of interest within each of the imputed datasets).[1] Because the intra-class correlation was found to be negligible, we used logistic regression for the final model using imputed data.

We checked whether the imputations were acceptable by comparing 1) the means, standard deviations, and plots of recorded and imputed values for continuous variables, and 2) the frequencies and percentages of recorded and imputed values for each level of categorical variables.

Analyses were done using SAS 9.3 and p-values less than 0.05 were considered statistically significant.

Auxiliary variables used in the imputation model

Variable	Questionnaire	Description of variable
**Psychological factors**		
Paternal affection	Health and Life Experiences Questionnaire (HLEQ)	Self-reported paternal affection using the Rossi scale.[2] The scale assessed items, such as, family composition, parental divorce and death, quality of relationship with the father and amount of affection received.
Mastery	Health and Life Experiences Questionnaire (HLEQ)	Self-reported using the Pearlin and Schooler Mastery Scale. Mastery is having a sense of control over one's life or the belief that one has control over future important life circumstances. It represents a coping resource that people use to manage or attenuate the impact of stressors, and this in turn, has an influence on health and health behaviours.[3,4]

For peer review only

Neuroticism	Health and Life Experiences Questionnaire (HLEQ)	Self-reported using the Eysenck Personality Inventory. A tendency towards experiencing negative, distressing emotions.[5]
**Sociodemographic factors**		
School age	Health and Lifestyle (HLQ) Questionnaire	Self-reported age when participant left school.
**Physical health**		
Systolic blood pressure	Baseline health check	Systolic blood pressure measured using an Accutorr noninvasive oscillometric blood pressure monitor; mean of two measurements in mmHg
Diastolic blood pressure	Baseline health check	Diastolic blood pressure measured using an Accutorr noninvasive oscillometric blood pressure monitor; mean of two measurements in mmHg
History of high cholesterol	Health and Lifestyle Questionnaire (HLQ)	Self-reported history of high cholesterol
History of psychiatric illness	Health and Lifestyle Questionnaire (HLQ)	Self-reported history of other psychiatric illness

The questionnaires used for these variables have been previously described in the methods.

The following auxiliary variables were not included in the imputation model, because they were not correlated with the variables in our model and were not good predictors of the missing status (following tests using Pearson's/Spearman's correlation coefficient and t-tests/chi-square tests): composite measure of maternal affection using the Rossi scale[2], and self-reported history of: migraine, benign tumours, psychiatric illness, and back pain.

For peer review only

References

1. Berglund P, Heeringa S. Multiple imputation of missing data using SAS. Cary, NC: *SAS Institute Inc*, 2014.
2. Rossi AS. Caring and doing for others: social responsibility in the domains of family, work, and community. Chicago: *University of Chicago Press*, 2001.
3. Pearlin LI, Menaghan EG, Lieberman MA, Mullan JT. The stress process. *J Health Soc Behav* 1981;22:337-56.
4. Pearlin LI, Schooler C. The structure of coping. *J Health Soc Behav* 1978;19:2-21.
5. Shipley BA, Weiss A, Der G, Taylor MD, Deary IJ. Neuroticism, extraversion, and mortality in the UK Health and Lifestyle Survey: a 21-year prospective cohort study.

Psychosom Med 2007;69:923-31 doi: 10.1097/PSY.0b013e31815abf83 [published Online First 8 November 2007].

For peer review only

Please see the article line numbers (column on the right) and the explanations provided.

STROBE Statement—checklist of items that should be included in reports of observational studies

	Item No	Recommendation	Line numbers within the article
**Title and abstract**	1	(*a*) Indicate the study's design with a commonly used term in the title or the abstract	7, 41
		(*b*) Provide in the abstract an informative and balanced summary of what was done and what was found	30-63
**Introduction**			
Background/rationale	2	Explain the scientific background and rationale for the investigation being reported	86-135
Objectives	3	State specific objectives, including any prespecified hypotheses	134-135
**Methods**			
Study design	4	Present key elements of study design early in the paper	139
Setting	5	Describe the setting, locations, and relevant dates, including periods of recruitment, exposure, follow-up, and data collection	140-144, 146-150, 157-158
Participants	6	(*a*) *Cohort study*—Give the eligibility criteria, and the sources and methods of selection of participants. Describe methods of follow-up<br>*Case-control study*—Give the eligibility criteria, and the sources and methods of case ascertainment and control selection. Give the rationale for the choice of cases and controls<br>*Cross-sectional study*—Give the eligibility criteria, and the sources and methods of selection of participants	140-144, 148-150, 152-153
		(*b*) *Cohort study*—For matched studies, give matching criteria and number of exposed and unexposed<br>*Case-control study*—For matched studies, give matching criteria and the number of controls per case	
Variables	7	Clearly define all outcomes, exposures, predictors, potential confounders, and effect modifiers. Give diagnostic criteria, if applicable	156-167, 169-185, 187-200, 202-218
Data sources/ measurement	8*	For each variable of interest, give sources of data and details of methods of assessment (measurement). Describe comparability of assessment methods if there is	There were three variables of interest in this study: area deprivation, sense of coherence, and generalized

For peer review only - http://bmjopen.bmj.com/site/about/guidelines.xhtml

		more than one group	anxiety disorder (187-200, 202-218, 156-167). The others are potential confounders – in the methods I list them all, indicate how they were assessed and mention that they were collected through the baseline, self-reported postal HLQ questionnaire as well as the HLEQ.
Bias	9	Describe any efforts to address potential sources of bias	245-246
Study size	10	Explain how the study size was arrived at	256-259
Quantitative variables	11	Explain how quantitative variables were handled in the analyses. If applicable, describe which groupings were chosen and why	In the methods, I mention how the variables were derived based on the raw data provided by participants in the questionnaires.<br><br>Dependent variable: 156-167<br>Area-level measure: 187-200, 233-235<br>Individual-level measures: 169-185, 246-252<br>Effect modifier: 202-218, 237-238<br><br>In the text, I mention that the categorization was done in accordance with the literature.
Statistical methods	12	(*a*) Describe all statistical methods, including those used to control for confounding	220-259
		(*b*) Describe any methods used to examine subgroups and interactions	I wanted to determine whether sense of coherence (SOC) was an effect modifier in the association between area deprivation and anxiety. I therefore compared the association between deprivation and anxiety in women with both high and low SOC. Before doing this, I examined the interaction between anxiety and SOC. This is described in the methods.
		(*c*) Explain how missing data were addressed	We indicated that this was a complete-case analysis. We also conducted multiple imputations for missing data.
		(*d*) *Cohort study*—If applicable, explain how loss to follow-up was addressed	Loss to follow-up was not a problem in this study.

For peer review only - http://bmjopen.bmj.com/site/about/guidelines.xhtml

Case-control study—If applicable, explain how matching of cases and controls was addressed<br>*Cross-sectional study*—If applicable, describe analytical methods taking account of sampling strategy	We were able to track down all participants using various means, unless they expressed that they wished to be removed from the mailing list. We elaborate on this in the manuscript.
(*e*) Describe any sensitivity analyses	We also conducted logistic regression in addition to GEE. This was discussed in the methods section.

For peer review only

For peer review only - http://bmjopen.bmj.com/site/about/guidelines.xhtml

**Results**			
Participants	13*	(a) Report numbers of individuals at each stage of study—eg numbers potentially eligible, examined for eligibility, confirmed eligible, included in the study, completing follow-up, and analysed	267-273, 276
		(b) Give reasons for non-participation at each stage	We do not have the reasons for non-participation, because these data were not collected when the study was initiated in 1993. There is some information comparing responders versus non-responders – Appendix I in our paper.
		(c) Consider use of a flow diagram	Flow diagram included in submission.
Descriptive data	14*	(a) Give characteristics of study participants (eg demographic, clinical, social) and information on exposures and potential confounders	278-280, 291-297; we provided characteristics for those with vs. without GAD, because we felt it was important to show the characteristics of those exposed vs. non-exposed (see also Table 1)
		(b) Indicate number of participants with missing data for each variable of interest	273-275
		(c) *Cohort study*—Summarise follow-up time (eg, average and total amount)	276
Outcome data	15*	*Cohort study*—Report numbers of outcome events or summary measures over time	278, 296
		Case-control study—Report numbers in each exposure category, or summary measures of exposure	
		Cross-sectional study—Report numbers of outcome events or summary measures	
Main results	16	(*a*) Give unadjusted estimates and, if applicable, confounder-adjusted estimates and their precision (eg, 95% confidence interval). Make clear which confounders were adjusted for and why they were included	Tables 2 and 3 contain unadjusted and progressively adjusted estimates. We also discussed the findings within the text, and provide odds ratios and 95% confidence intervals (ex. 330-345). We included the confounders based on the literature – we mention this in the paper. As per strobe, we included this information in the methods section; and we omitted repeating this in the results section to reduce redundancy. However, if the editor would like us to repeat this information in the results, we are happy to

For peer review only - http://bmjopen.bmj.com/site/about/guidelines.xhtml

For peer review only

			do so.
		(*b*) Report category boundaries when continuous variables were categorized	The age cut-offs are provided. In regards to the Townsend index, the methods section states that those below and above the cut-point of zero were compared. In regards to the SOC, those above and below the cut-point of 2 were split.
		(*c*) If relevant, consider translating estimates of relative risk into absolute risk for a meaningful time period	
Other analyses	17	Report other analyses done—eg analyses of subgroups and interactions, and sensitivity analyses	Multiple imputations for missing data were carried out and results are reported. We also reported the results for logistic regression, as well as findings for the test of interaction between area deprivation and SOC.
**Discussion**			
Key results	18	Summarise key results with reference to study objectives	357-360, 365-380
Limitations	19	Discuss limitations of the study, taking into account sources of potential bias or imprecision. Discuss both direction and magnitude of any potential bias	406-445
Interpretation	20	Give a cautious overall interpretation of results considering objectives, limitations, multiplicity of analyses, results from similar studies, and other relevant evidence	494-510 (We also have a section comparing our study results to those of others: 450-482, as well as a section on potential mechanisms explaining our findings: 484-492)
Generalisability	21	Discuss the generalisability (external validity) of the study results	428-430
**Other information**			
Funding	22	Give the source of funding and the role of the funders for the present study and, if applicable, for the original study on which the present article is based	521-522

*Give information separately for cases and controls in case-control studies and, if applicable, for exposed and unexposed groups in cohort and cross-sectional studies.

Note: An Explanation and Elaboration article discusses each checklist item and gives methodological background and published examples of transparent reporting. The STROBE checklist is best used in conjunction with this article (freely available on the Web sites of PLoS Medicine at http://www.plosmedicine.org/, Annals of Internal Medicine at http://www.annals.org/, and Epidemiology at http://www.epidem.com/). Information on the STROBE Initiative is available at www.strobe-statement.org.

For peer review only - http://bmjopen.bmj.com/site/about/guidelines.xhtml
